# Inhibition of sphingolipid de novo synthesis counteracts muscular dystrophy

**DOI:** 10.1126/sciadv.abh4423

**Published:** 2022-01-28

**Authors:** Pirkka-Pekka Laurila, Peiling Luan, Martin Wohlwend, Nadège Zanou, Barbara Crisol, Tanes Imamura de Lima, Ludger J. E. Goeminne, Hector Gallart-Ayala, Minho Shong, Julijana Ivanisevic, Nicolas Place, Johan Auwerx

**Affiliations:** 1 Laboratory of Integrative Systems Physiology, École Polytechnique fédérale de Lausanne (EPFL), Lausanne, Switzerland.; 2 Institute of Sport Sciences, Department of Physiology, Faculty of Biology-Medicine, University of Lausanne, Lausanne, Switzerland.; 3 Metabolomics Platform, Faculty of Biology and Medicine, University of Lausanne (UNIL), Lausanne, Switzerland.; 4 Research Center for Endocrine and Metabolic Diseases, Chungnam National University Hospital, Chungnam National University School of Medicine, Daejeon, Republic of Korea.; 5 Department of Medical Science, Chungnam National University School of Medicine, Daejeon, Republic of Korea.

## Abstract

Duchenne muscular dystrophy (DMD), the most common muscular dystrophy, is a severe muscle disorder, causing muscle weakness, loss of independence, and premature death. Here, we establish the link between sphingolipids and muscular dystrophy. Transcripts of sphingolipid de novo biosynthesis pathway are up-regulated in skeletal muscle of patients with DMD and other muscular dystrophies, which is accompanied by accumulation of metabolites of the sphingolipid pathway in muscle and plasma. Pharmacological inhibition of sphingolipid synthesis by myriocin in the *mdx* mouse model of DMD ameliorated the loss in muscle function while reducing inflammation, improving Ca^2+^ homeostasis, preventing fibrosis of the skeletal muscle, heart, and diaphragm, and restoring the balance between M1 and M2 macrophages. Myriocin alleviated the DMD phenotype more than glucocorticoids. Our study identifies inhibition of sphingolipid synthesis, targeting multiple pathogenetic pathways simultaneously, as a strong candidate for treatment of muscular dystrophies.

## Teaser

Sphingolipids accumulate in dystrophic muscle.

## INTRODUCTION

Muscular dystrophies are a heterogeneous group of inherited genetic diseases characterized by loss of membrane integrity and muscle weakness and degeneration. Duchenne muscular dystrophy (DMD) is the most common muscular dystrophy affecting 1 in 3500 newborn boys and is caused by one of the thousands of identified mutations in DMD gene (*1*) encoding dystrophin, a scaffolding protein that supports muscle structure by anchoring the cytoskeleton with the sarcolemma. Patients diagnosed with DMD often experience premature death due to cardiac or respiratory failure following protracted muscle degeneration in the heart and diaphragm (*1*). Currently, there is no cure for DMD. Despite recent advances in CRISPR-Cas9–mediated gene editing (*2*), the high allelic heterogeneity of the disease and the safety concerns of recent clinical trials (*3*) present challenges to gene therapy, suggesting that pharmacological approaches are required to complement these therapies and to help manage patients with mutations unsuitable for gene editing technologies.

Numerous biological pathways contribute to the pathophysiology of DMD. Compromised membrane integrity, aberrant calcium homeostasis, chronic inflammation, fibrosis, and impaired tissue remodeling are pathological hallmarks of the disease (*4*). Although efforts to target these pathways individually, including restoration of Ca^2+^ homeostasis with Ca^2+^ channel blockers (*5*) and muscle mass with anabolic steroids (*6*), have been subject to clinical trials, they have not produced clinical benefits relevant for the overall course of the disease. Glucocorticoids with their immunosuppressive effects are currently the only pharmacological treatment for DMD incorporated in the treatment guidelines (*7*–*9*), but their efficacy is suboptimal, and their side effects are considerable. Optimally, treatment strategies would not only target one but several pathways involved in DMD pathogenesis. The high prevalence and the debilitating nature of the disease call for urgent efforts to develop new disease-modifying treatment strategies for DMD.

Sphingolipids are bioactive lipids with pleiotropic functions, including in inflammation, fibrosis, and cell death (*10*). Ceramides serve as the central intermediate of sphingolipid metabolism; they can be synthesized from serine and palmitate through the sphingolipid de novo synthesis pathway (*10*). Increased ceramide levels have been implicated in many diseases, including Alzheimer’s disease (*11*), cardiovascular disease (*12*), and diabetes (*13*). Furthermore, it was shown that skeletal muscles also accumulate sphingolipids in obesity (*14*) and depletion of muscular sphingolipids ameliorates obesity-induced insulin resistance and improves glucose homeostasis in animals fed with high-fat diet (*15*). While intensive research efforts have focused on exploring the role of sphingolipid depletion in treating metabolic disorders, the role of sphingolipid metabolism in muscular dystrophy still remains unproven. DMD shares many biological pathways with metabolic disorders, calling for testing whether inhibition of sphingolipid de novo synthesis pathway, having diverse effects in cellular physiology (*10*), could restore muscle function in DMD.

In the present study, we establish the link between muscular dystrophies and sphingolipid metabolism. We demonstrate that metabolic intermediates of the sphingolipid de novo synthesis pathway accumulate in mouse models of muscular dystrophy, accompanied by the up-regulation of enzymes involved in sphingolipid de novo synthesis at both mRNA and protein levels. Reducing sphingolipid de novo synthesis by myriocin, an inhibitor of serine palmitoyltransferase (SPT), the first and rate-limiting enzyme of the sphingolipid biosynthesis pathway, counteracts DMD-related loss in muscle function in the *mdx* mouse model of muscular dystrophy. Blocking sphingolipid synthesis stabilizes muscular Ca^2+^ turnover, reverses diaphragmatic and cardiac fibrosis, and attenuates DMD-associated muscle inflammation by directing macrophage polarization toward the anti-inflammatory state. Last, we demonstrate in *mdx* mice the superiority of sphingolipid synthesis inhibition over glucocorticoids, used in standard DMD care. Given that pharmacological sphingolipid reduction alleviated dystrophic symptoms and reversed multiple pathophysiological hallmarks of DMD, we propose that inhibition of sphingolipid de novo synthesis could be an attractive therapeutic strategy to treat muscular dystrophies.

## RESULTS

### Sphingolipid de novo synthesis pathway is up-regulated in muscular dystrophy

**Fig. 1.**
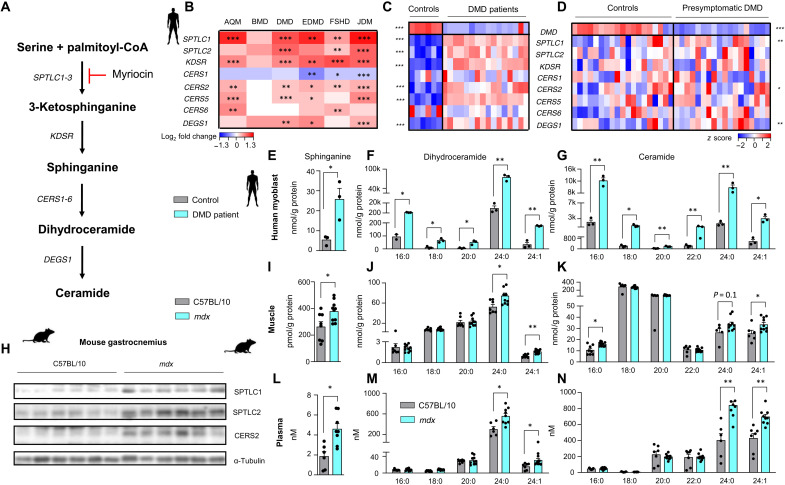
Sphingolipid de novo synthesis pathway is up-regulated in human muscular dystrophy and mouse model of DMD. (**A**) Sphingolipid de novo synthesis pathway. CoA, coenzyme A. (**B**) Transcript abundance of enzymes of sphingolipid de novo synthesis pathway in human skeletal muscle biopsies from patients affected with different muscle diseases (GSE3307). Data are shown as log_2_ fold change of affected patients (*n* for each disease is provided below) relative to healthy controls (*n* = 36) and Benjamini-Hochberg-adjusted FDRs (BH FDRs) obtained from GSE3307. AQM (*n* = 10), BMD (*n* = 10), DMD (*n* = 20), EDMD (*n* = 9), FSHD (*n* = 28), and JDM (*n* = 42). (**C**) Transcript abundance of enzymes involved in the sphingolipid de novo synthesis pathway in human patients affected by DMD (*n* = 16) and healthy controls (*n* = 6). Dataset GSE38417. (**D**) Transcript abundance of enzymes involved in the sphingolipid de novo synthesis pathway in presymptomatic patients diagnosed with DMD (*n* = 20) and healthy controls (*n* = 17). Dataset GSE6011. For (C) and (D), data are given as *z* scores, and *P* values are obtained using Student’s two-tailed *t* test adjusting for the BH FDR over these nine genes. Sphinganine (**E**), dihydroceramide (**F**), and ceramide (**G**) levels in human myoblasts derived from patients with DMD. (**H**) Western blot analysis of enzymes of the sphingolipid de novo synthesis pathway in gastrocnemius muscles from C57BL/10SnJ mice and *mdx* mice treated with either dimethyl sulfoxide (DMSO) or myriocin. Sphinganine (**I**), dihydroceramide (**J**), and ceramide (**K**) levels in quadriceps muscle (I to K) and plasma (**L** to **N**) from *mdx* mice. For (E) to (N), statistical significance is calculated using Student’s two-tailed *t* test with BH adjustment for FDR. All data are shown as means ± SEM. *P < 0.05, **P < 0.01, and ***P < 0.001.

The sphingolipid de novo synthesis pathway produces ceramides and other sphingolipids using fatty acids and amino acids as substrates. SPT is the first and rate-limiting enzyme of the pathway, generating 3-ketosphinganine, which is rapidly converted to sphinganine (Fig. 1A). The conversion of sphinganine to dihydroceramide is achieved by one of the six mammalian ceramide synthases (CERS1 to CERS6), of which each has specificity for acyl chains of different lengths and which are differentially expressed across tissues and cell types.

To evaluate the role of the sphingolipid de novo synthesis pathway in muscular dystrophies, we compared the transcript abundance of enzymes of the pathway in a publicly available dataset featuring muscle biopsies derived from patients diagnosed with different muscular dystrophies to healthy controls (*16*). Across multiple muscle diseases, including DMD, Becker muscular dystrophy (BMD), Emery-Dreifuss muscular dystrophy (EDMD), and facioscapulohumeral muscular dystrophy (FSHD), we found an almost universal up-regulation of sphingolipid biosynthetic enzymes, with the exception of *CERS1* (Fig. 1B). These observations demonstrate the robust involvement of the sphingolipid de novo synthesis pathway in human muscular dystrophies.

DMD is the most common muscular dystrophy, and along with acute quadriplegic myopathy (AQM) and juvenile dermatomyositis (JDM), it presented the most notable up-regulation of sphingolipid de novo synthesis pathway in muscle (Fig. 1B). We replicated these findings in another dataset of patients with DMD (1 to 8 years of age) (*17*) in which subunits of SPT (SPTLC1 and SPTLC2) along with CERS2, the ceramide synthase responsible for production of very-long-chain sphingolipids, were consistently induced (Fig. 1C). In an additional dataset (*18*) consisting of boys with confirmed genetic diagnosis of DMD but in which biopsies were obtained before the onset of symptoms, we also observed an up-regulation of transcripts of the same genes in the sphingolipid de novo synthesis pathway (Fig. 1D). The relative abundance of sphingolipid biosynthetic enzymes was more pronounced in symptomatic than presymptomatic patients, suggesting that the sphingolipid biosynthetic pathway correlates with disease severity.

To study whether transcriptional up-regulation of the sphingolipid de novo synthesis reflects the abundance of the metabolic intermediates of the pathway, we performed mass spectrometry–based sphingolipid analysis from human primary myoblasts isolated from patients with DMD and controls. The content of sphinganine, dihydroceramides, and ceramides was increased in myoblasts derived from patients with DMD (Fig. 1, E to G), indicating that transcriptional up-regulation of sphingolipid biosynthetic process translates into increased metabolite levels of the pathway.

The *mdx* mice, a mouse model widely used in DMD research, have a point mutation in the dystrophin encoding *Dmd* gene, similar to human patients with DMD. This genetic defect leads to the production of a small, nonfunctional dystrophin protein. As compared to wild-type (WT) C57BL/10SnJ mice, the abundance of enzymes of the sphingolipid de novo synthesis pathway was robustly elevated at the protein level in gastrocnemius muscle of *mdx* mice (Fig. 1H). Accordingly, the levels of intermediates of the sphingolipid de novo synthesis pathway, including sphinganine and certain dihydroceramides and ceramides (Fig. 1, I to K), were elevated in skeletal muscle.

Muscular dystrophy in *mdx* mouse is characterized by rapid onset of muscle fiber degeneration with intense inflammatory infiltrate present since weaning (*19*). This process peaks at the age of 4 weeks and is followed by effective muscular regeneration phase. To study whether muscle sphingolipid content is associated with disease severity of muscular dystrophy, we measured sphingolipid levels in 4-week-old (representing muscular degeneration) and 10-week-old (representing muscular regeneration) *mdx* mice, comparing them with WT mice. Sphingolipid levels were higher in 4-week-old *mdx* as compared to 10-week old *mdx* mice (fig. S1, A and B). Thus, muscle sphingolipid levels appear to correlate with disease severity, in line with transcript abundance in human muscle biopsies (Fig. 1, C and D).

In particular, the levels of very-long-chain sphingolipids, which are synthesized by CERS2, were increased. The increase in the sphingolipid de novo synthesis pathway was also reflected in the plasma of *mdx* mice (Fig. 1, L to N), where we observed a robust increase in sphinganine levels and, as in muscle tissue, an increase in very-long-chain sphingolipids. In general, our analyses show that the sphingolipid synthesis pathway is up-regulated in muscular dystrophies.

### Inhibition of sphingolipid de novo synthesis restores muscle function in mouse model of DMD

**Fig. 2.**
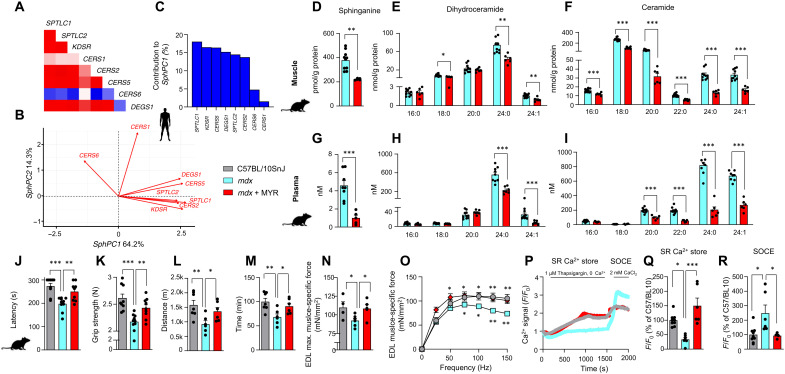
Inhibition of sphingolipid de novo synthesis improves muscle function in *mdx* mice. (**A**) Correlation of transcripts of sphingolipid de novo synthesis pathway in skeletal muscle of symptomatic DMD patients and controls (E-GEOD-38417). (**B**) Factor loading plot (biplot) showing the effects of the enzymes of sphingolipid de novo synthesis pathway on two first PCs (SphPC1 and SphPC2) in human symptomatic patients with DMD and controls. (**C**) Contribution of each transcript to the SphPC1 in human skeletal muscle in symptomatic patients with DMD and controls. Skeletal muscle sphinganine (**D**), dihydroceramide (**E**), and ceramide (**F**) levels in C57BL/10SnJ and *mdx* mice treated with DMSO or myriocin (MYR). For (D) to (F), statistical significance is calculated using Student’s two-tailed *t* test with BH adjustment for FDR. Plasma sphinganine (**G**), dihydroceramide (**H**), and ceramide (**I**) levels. For (G) to (I), statistical significance is calculated using Student’s two-tailed *t* test with BH adjustment for FDR. For (D) to (I), the samples for the “*mdx*” group are the same as for Fig. 1. (**J** to **O**) Comparison of maximal latency of rotarod test (J), grip strength (K), maximal running distance (L), and time (M) on a treadmill between C57BL/10 Snj, *mdx* mice, and *mdx* mice treated with myriocin. (N) Maximal specific isometric force in EDL developed during a 300-ms tetanus stimulation. (O) EDL muscle force-frequency relationship. (**P**) Representative traces of cytosolic Ca^2+^ transients in isolated FDB muscle fibers upon 1 μM thapsigargin stimulation (SR Ca^2+^ store) and after 2 mM CaCl_2_ (SOCE). (**Q**) Grouped data of Ca^2+^ amplitude (SR Ca^2+^ store) upon 1 μM thapsigargin stimulation as a percentage of C57/BL10. (**R**) SOCE upon 1 μM thapsigargin stimulation as a percentage of C57/BL10. All data are shown as means ± SEM. Statistical significance is calculated using Student’s two-tailed *t* test with BH adjustment for FDR. *P < 0.05, **P < 0.01, and ***P < 0.001.

Further analysis of human skeletal muscle transcript profile of biopsies from patients diagnosed with DMD and controls revealed a strong positive correlation between different transcripts of the sphingolipid de novo biosynthesis pathway (Fig. 2A), except for *CERS1* and *CERS6*. Following the strong correlations between transcripts of the pathway, we conducted principal components analysis of gene expression on the sphingolipid de novo biosynthesis pathway in humans. The first principal component (PC) of the expression of different enzymes of the sphingolipid de novo synthesis pathway (SphPC1) explained 64.2% of the variance in human skeletal muscle (Fig. 2B and fig. S2A). Of the transcripts of the pathway, *SPTLC1* contributed the most, accounting for 18% of SphPC1 variability in humans (Fig. 2C). These results suggest that the sphingolipid de novo biosynthesis pathway is under tightly coordinated transcriptional control.

We next asked the question whether pharmacological inhibition of the sphingolipid synthesis pathway could restore muscle function and ameliorate symptoms of DMD in a mouse model. To block sphingolipid synthesis, we treated *mdx* mice for 6 months with intraperitoneal injections of myriocin (0.4 mg/kg, three times per week), an inhibitor of SPT, an enzyme encoded by the subunits *SPTLC1* and *SPTLC2*. As expected, myriocin reduced sphinganine, dihydroceramide, and ceramide levels both in the muscle (Fig. 2, D and E) and plasma (Fig. 2, G to I), confirming the desired effect of the compound on sphingolipid synthesis. As hepatotoxic effects of myriocin in rats were reported in a previous study (*20*), we measured plasma levels of alanine aminotransferase and aspartate aminotransferase in myriocin-treated mice. The plasma levels of these enzymes did not differ from untreated *mdx* mice (fig. S2, B and C).

Myriocin counteracted DMD-related loss in muscle function. Relative to WT mice, *mdx* mice performed worse in rotarod, grip strength, and uphill running tests (Fig. 2, J to M) assessing coordination, strength, and endurance of the animals, respectively. Myriocin improved the performance of *mdx* mice in these tests. Furthermore, while *mdx* mice displayed lower maximal specific force and a downward shift of force-frequency relationship of extensor digitorum longus (EDL) as compared to WT mice, myriocin-treated mice had increased maximal EDL-specific force and an upward shift of force-frequency relationship without alteration of the mechanical properties of the muscles (similar force values when forces are normalized by the maximal force generation capacity) (Fig. 2, N and O). There was also a tendency for fatigue resistance in myriocin-treated mice (fig. S2, D and E), while eccentric contraction was similar between *mdx* mice and *mdx* mice treated with myriocin (fig. S2F). These data demonstrate that myriocin promotes functional improvement in DMD.

Abnormal Ca^2+^ influx and release are hallmarks of DMD (*21*), primarily owing to the aberrant function of Ca^2+^- handling proteins. Dysregulation of store-operated calcium channels has also been reported in DMD (*22*). To study the effects of inhibition of sphingolipid synthesis on Ca^2+^ homeostasis, we dissociated single flexor digitorum brevis (FDB) muscle fibers from dimethyl sulfoxide (DMSO) and myriocin-treated *mdx* mice along with C57BL/10SnJ controls and stimulated them with thapsigargin, caffeine, or histamine to release Ca^2+^ from the sarcoplasmic reticulum (SR). We then also assessed store-operated Ca^2+^ entry (SOCE) by adding exogenous Ca^2+^ to the fibers. The response to thapsigargin-, caffeine-, or histamine-induced SR Ca^2+^ release was significantly blunted in *mdx* mice as compared to WT mice (Fig. 2, P to Q, and fig. S2, G to I). Myriocin restored the SR Ca^2+^ release in *mdx* muscle to the levels of WT controls under all stimulation conditions (Fig. 2, P to Q, and fig. S2, G to I), suggesting rescued Ca^2+^ stores in myriocin-treated *mdx* and possibly explaining the improvements in muscle strength and force production in myriocin-treated *mdx* mice (Fig. 2, K and N). Notably, the pumping of Ca^2+^ from the cytosol to the SR after caffeine stimulation (Ca^2+^ reuptake) was impaired in *mdx* but not myriocin-treated *mdx* mice relative to WT mice (fig. S2J). SOCE was impaired in *mdx* mice (Fig. 2R), likely reflecting deteriorated cellular membranes in *mdx* mice. Myriocin enhanced SOCE in muscle fibers (Fig. 2R), pointing toward potential improvements in membrane integrity. Overall, our results suggest that myriocin improves myocellular Ca^2+^ homeostasis in *mdx* mice. These findings support the functional improvements induced by myriocin in the skeletal muscle.

### Myriocin protects membrane integrity and structural homeostasis of skeletal muscle

**Fig. 3.**
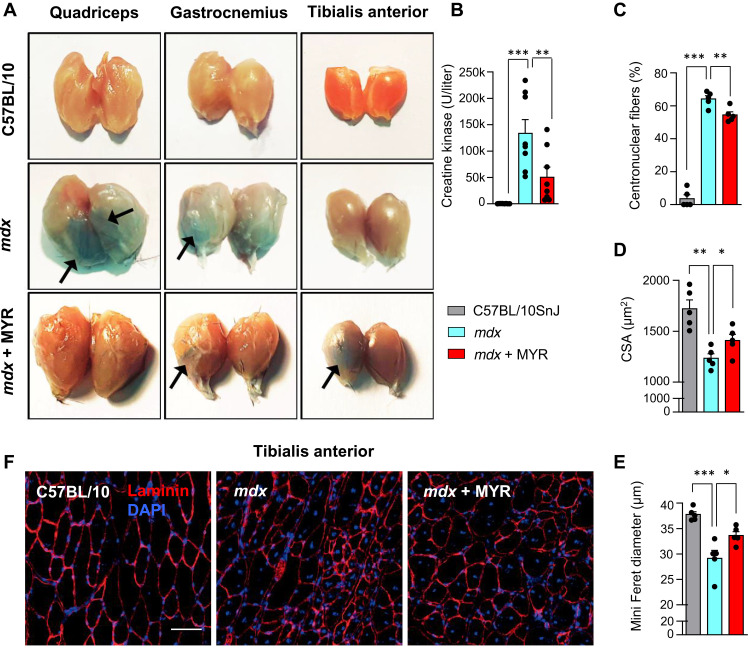
Inhibition of sphingolipid de novo synthesis restores integrity of muscle structures and improves muscle function of *mdx* mice. (**A**) EBD staining of quadriceps, gastrocnemius, and tibialis anterior (TA) muscles. (**B**) Plasma CK levels following eccentric exercise (*n* = 8/8/8). (**C**) Proportion of fibers with centralized nuclei in C57BL/10 Snj, DMSO- and myriocin-treated *mdx* mice. Mean CSA (**D**) and mean of fiber minimal Feret diameter (**E**) in WT mice, *mdx* mice, and *mdx* mice treated with myriocin [for (C) to (E), *n* = 5/5/5]. (**F**) Laminin staining of TA muscle. Scale bar, 50 μm. All data are shown as means ± SEM. Statistical significance is calculated using Student’s two-tailed *t* test with BH adjustment for FDR. *P < 0.05, **P < 0.01, and ***P < 0.001.

Compromised membrane integrity of sarcolemma is a hallmark of DMD (*23*), as dystrophin is required to maintain the mechanical integrity of the plasma membrane (*24*). Membrane disruptions in DMD are large enough to permit release of intracellular proteins, such as creatine kinase (CK), and uptake of vital dyes, such as Evans blue (EBD) (*25*). In *mdx* mice, we observed compromised membrane integrity, as evidenced by increased uptake of EBD into skeletal muscle (Fig. 3A), whereas myriocin reduced skeletal muscle EBD uptake (Fig. 3A). After eccentric exercise during downhill running, *mdx* mice displayed increased plasma CK levels, while attenuated CK release was observed in myriocin-treated mice (Fig. 3B). These findings indicate that myriocin counteracts the loss of membrane integrity in DMD.

Many different muscle diseases (*26*), including DMD (*27*), are characterized by nuclei prominently located within the center of individual muscle fibers. While *mdx* mice displayed far more centrally located nuclei than C57BL/10SnJ mice, this tendency was somewhat attenuated in myriocin-treated mice (Fig. 3C). In line with previous studies, muscle fibers of *mdx* mice exhibited smaller cross-sectional area (CSA) (Fig. 3D) than WTs in addition to reduced Feret diameter (Fig. 3, E and F), which were both improved by myriocin. These findings demonstrate that myriocin alleviates DMD-related morphological alterations in skeletal muscle.

### Myriocin alleviates inflammation and shifts macrophage balance toward anti-inflammatory state

**Fig. 4.**
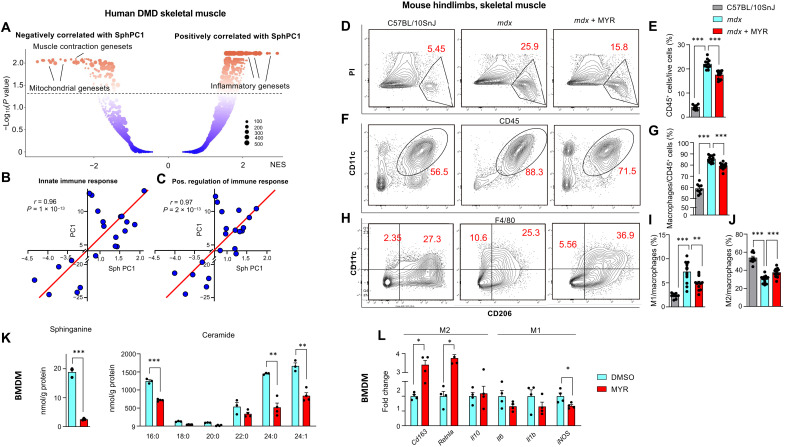
Inhibition of sphingolipid de novo synthesis alleviates DMD-associated inflammatory response. (**A**) GSEA of transcripts correlating with SphPC1 in human skeletal muscle biopsies of patients with DMD and controls in E-GEOD-38417 dataset (*n* = 22). Correlation of SphPC1 with the first PC of the GO: innate immune response. NES, normalized effect size. (**B**) and GO: positive regulation of immune response (**C**), respectively, in skeletal muscle of patients with DMD and controls (E-GEOD-38417). (**D**) Representative images of FACS contour plot of CD45^+^PI^+^ live cells and (**E**) corresponding quantification of CD45^+^ cells normalized to the number of total propidium iodide (PI)^+^ live cells. (**F**) Representative images of FACS contour plot of CD11b^+^F4/80^+^ macrophages and FACS-based quantification (**G**) of CD11b^+^F4/80^+^ macrophages, normalized to the number of total immune cells. (**H**) Representative images of FACS contour plot of distinct macrophage subpopulations and corresponding quantification of M1 (**I**) and M2 (**J**) cells normalized to the total number of macrophages (*n* = 9/13/12). (**K**) Sphinganine (left) and ceramide (right) levels in DMSO- and myriocin-treated primary BMDMs isolated from *mdx* mice. BMDMs were stimulated with IL-4 (100 ng/ml) for 48 hours in the absence or presence of myriocin. Statistical significance is calculated using Student’s two-tailed *t* test with BH adjustment for FDR. *n* = 3/4 per condition. (**L**) mRNA levels of M1 and M2 macrophage markers in primary BMDMs isolated from *mdx* mice. BMDMs were stimulated with IL-4 (100 ng/ml) or 48 hours in the absence or presence of myriocin. *n* = 4 per condition. For (L), *P* values were calculated using Student’s two-tailed *t* test on log-transformed values with BH adjustment for FDR. For other panels, statistical significance is calculated using Student’s two-tailed *t* test with BH adjustment for FDR. All data are shown as means ± SEM. *BH FDR < 0.05, **BH FDR < 0.01, and ***BH FDR < 0.001.

To identify biological pathways whose modulation could be associated with the improved functional performance upon myriocin treatment, we studied the skeletal muscle transcript profiles of patients with DMD and controls in an unbiased manner. We calculated the Pearson correlation coefficient (*r*) between SphPC1 (the first principal component of the sphingolipid de novo synthesis pathway, see Fig. 2B) and every identified transcript from the genome-wide expression data and performed gene set enrichment analysis (GSEA) ranking all transcripts in descending order of their Pearson’s *r* with SphPC1. The most positively correlated pathways with SphPC1 were related to inflammation (Fig. 4A), including “gene ontology (GO) positive regulation of immune response”, followed by “GO cell adhesion” and “GO innate immune response” (table S1). In addition, the first PCs of these pathways were positively correlated with SphPC1: GO positive regulation of immune response, *r* = 0.967, *P* = 2 × 10^−13^ (Fig. 4B); and GO innate immune response, *r* = 0.968, *P* = 1 × 10^−13^ (Fig. 4C).

Chronic activation of the innate immune system and associated inflammation are observed in patients with DMD soon after birth before the onset of clinical symptoms (*28*). Macrophages are integral players in the innate immune response, and classically activated M1 macrophages play a strong role in muscle injury. Macrophage infiltration is the most prominent immune feature observed in the muscle of *mdx* mice (*29*), and macrophage depletion has been shown to reduce muscle lesions (*30*).

Myriocin effectively reduced general inflammation in skeletal muscle of *mdx* mice, as evidenced by the decreased number of CD45^+^ immune cells (Fig. 4, D and E). Consistent with reported infiltration of macrophages into skeletal muscle in patients with DMD (*31*), we observed elevated macrophage count in skeletal muscle of *mdx* mice (Fig. 4, F and G). The abundance of skeletal muscle macrophages was reduced by myriocin treatment.

Macrophages are capable of shifting to either predominantly proinflammatory M1 or predominantly anti-inflammatory M2 states (*32*), and distinct subpopulations of macrophages can promote muscle injury or repair in muscular dystrophy (*33*). We used fluorescence-activated cell sorting (FACS) to characterize the proportion of M1 macrophages, expressing CD11c, and M2 macrophages, expressing CD206. We observed an imbalance of M1 macrophages in skeletal muscle of *mdx* mice as compared to WT controls (Fig. 4, H and I). Myriocin reversed this profile, shifting the balance of skeletal muscle macrophage population toward M2 (Fig. 4, H to J). To evaluate the direct effect of myriocin on macrophages from dystrophic mice, we isolated bone marrow–derived primary monocytes from *mdx* mice, differentiated them into macrophages, and exposed them to interleukin-4 (IL-4), a cytokine known to drive the activation of M2 macrophages. Notably, bone marrow–derived macrophages (BMDMs) isolated from *mdx* mice exhibited elevated levels of the intermediates of the sphingolipid de novo synthesis pathway, including sphinganine, dihydroceramides, and very-long-chain ceramides (fig. S3, A and C), suggesting a global up-regulation sphingolipid de novo synthesis pathway across tissues. Macrophages cultured in myriocin-containing medium exhibited lower sphingolipid levels (Fig. 4K) and higher expression of markers of M2 macrophages (e.g., *Cd136* and *Retnla*) in the presence of similar or modestly reduced (i.e., *iNOS*) M1 markers (Fig. 4L). These findings indicate that myriocin affects the macrophage subpopulation in dystrophic skeletal muscle and has a cell-autonomous effect on primary macrophages derived from *mdx* mice.

### Myriocin reduces fibrosis in skeletal muscle, heart, and diaphragm

**Fig. 5.**
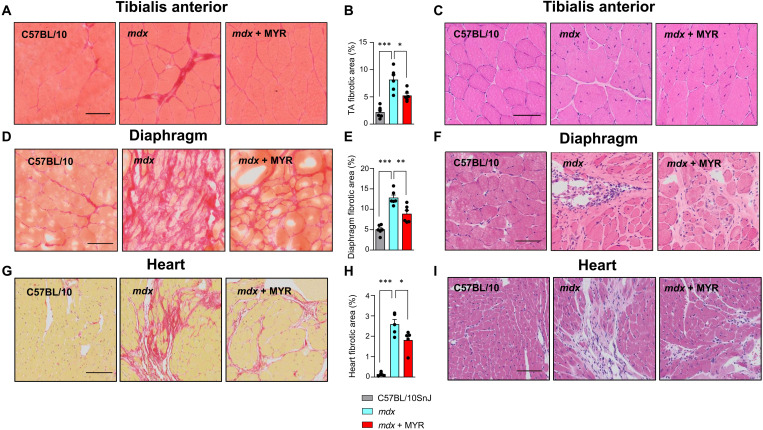
Myriocin improves fibrosis in *mdx* mice. Representative images from Picrosirius red staining (**A**), quantification (**B**), and representative images of hematoxylin and eosin (H&E) staining (**C**) of TA from control C57BL/10JSnj mice and *mdx* mice with and without myriocin treatment. Scale bars, 50 μm. Representative images from Picrosirius red staining (**D**), quantification (**E**), and representative images of H&E staining (**F**) of diaphragm from control mice and *mdx* mice with and without myriocin treatment. Scale bars, 50 μm. Representative images from Picrosirius red staining (**G**), quantification (**H**), and representative images of H&E staining (**I**) of heart (left ventricle) from control mice and *mdx* mice with and without myriocin treatment. Scale bars, 100 μm. *n* = 6/6/6. All data are shown as mean ± SEM. Statistical significance is calculated using Student’s two-tailed *t* test with BH adjustment for FDR. *BH FDR < 0.05, **BH FDR < 0.01, and ***BH FDR < 0.001.

Muscle fibrosis is a hallmark of DMD (*34*). Different biological processes can drive fibrosis in DMD, including inflammation (*35*), and muscle fibrosis can impair tissue homeostasis and promote muscle weakness. Relative to WT controls, *mdx* mice exhibited increased skeletal muscle fibrosis. Myriocin reduced fibrosis, as evidenced by Picrosirius staining (Fig. 5, A to C).

Progressive muscle weakness in DMD causes the loss of motor skills and ultimately paralysis, and respiratory failure and cardiomyopathy are observed in later stages of the disease (*36*). The *mdx* diaphragm well recapitulates a pattern of degeneration, fibrosis, and the severe functional deficits comparable to that of the diaphragm in human DMD (*37*). The diaphragms of *mdx* mice displayed elevated sphingolipid levels (fig. S4A). Extensive fibrosis was observed in 34-week-old *mdx* mice relative to WT controls, and this hallmark of imminent respiratory failure was substantially attenuated by myriocin (Fig. 5, D to F).

The *mdx* mouse also develops cardiomyopathy progressively with age yet a milder form than observed in human DMD (*38*). Cardiac fibrosis, defined as the excessive accumulation of extracellular matrix such as collagens in the heart (*39*), is readily observed in *mdx* mice (*40*). Increased levels of sphingolipids were observed in the heart of *mdx* mice (fig. S4B). In line with previous studies, we observed increased cardiac fibrosis in *mdx* mice as compared to WT mice, and myriocin effectively reduced fibrosis in the heart (Fig. 5, G to I). Our results thus demonstrate that myriocin confers substantial morphological benefits to the diaphragm and heart of *mdx* mice, vital organs severely affected in DMD.

Fibro/adipogenic precursors (FAPs) are nonmyogenic progenitors that can undergo fibrogenic or adipogenic differentiation (*41*). They are marked by surface expression of platelet-derived growth factor receptor α (PDGFRα). The FAPs have been reported to substantially contribute to DMD-induced fibrosis (*42*). Notably, an abnormal presence of FAPs is observed in DMD, correlating with the severity of the disease (*43*), and impaired FAP clearance has been shown to result in muscle loss and fibrosis (*44*). Consistent with fibrotic changes, *mdx* mice displayed more PDGFRα^+^ cells in diaphragm, and myriocin reduced the accumulation of PDGFRα^+^ cells (fig. S5A). Furthermore, increased number of PDGFRα^+^ cells was also observed in the tibialis anterior (TA) muscle of *mdx* mice, and their deposition was reduced by myriocin (fig. S5B). Together, our findings demonstrate that myriocin has both anti-inflammatory and antifibrotic effects on dystrophic muscle, consistent with improved muscle function.

### Myriocin provides additional health benefits to glucocorticoid treatment

Although no cure for DMD currently exists, glucocorticoids represent the standard care for DMD, and their use in patients with DMD is associated with prolonged mobility and muscle strength (*45*). Glucocorticoids, however, cause many untoward side effects, including weight gain and growth delay. Previous studies in *mdx* mice have shown some benefits of glucocorticoid treatment on muscle regeneration (*46*) and morphology (*47*, *48*), yet daily administration of glucocorticoids has resulted in weight loss, reduced strength, and increased fibrosis in the heart (*49*). This problem was overcome in a recent study demonstrating that weekly administration of glucocorticoids improved fitness when compared to daily dosing, which impaired muscle performance in *mdx* mice (*50*). To evaluate the relative efficacy of myriocin to weekly glucocorticoid administration and to assess whether their combined administration could deliver additional fitness benefits, we treated *mdx* mice with monotherapy of deflazacort and myriocin and their respective combination for 2 months in accordance with the previous study (*50*).

**Fig. 6.**
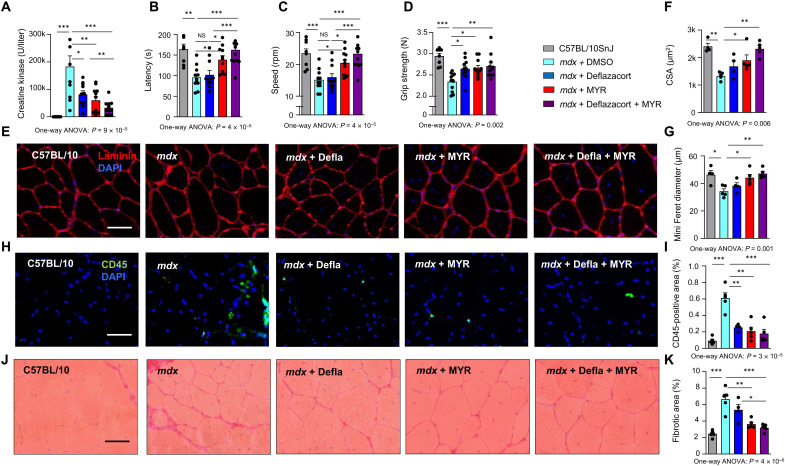
Myriocin and deflazacort alleviate muscular dystrophy in an additive manner. (**A**) Plasma CK levels following eccentric exercise in WT C57BL/10JSnj mice, *mdx* mice, and *mdx* mice treated with deflazacort, myriocin, and their combination therapy. Maximal latency (**B**) and speed (**C**) in the rotarod test and grip strength (**D**). *n* = 8/11/11/11/11. (**E**) Representative images of laminin staining of TA muscle. Scale bar, 50 μm. Mean CSA (**F**) and mean of fiber minimal Feret diameter (**G**) in TA muscle. Representative images and quantification of CD45 immunofluorescence (**H** and **I**) and Sirius red (**J** and **K**) staining from TA of WT mice, *mdx* mice, and *mdx* mice treated with deflazacort, myriocin, and their combination therapy. *n* = 4 to 5 per group. All data are shown as means ± SEM. *P < 0.05, **P < 0.01, and ***P < 0.001 for Tukey’s post hoc test. *P* values for ANOVA are provided. NS, not significant.

After the treatment, we performed downhill running and measured CK levels following eccentric exercise. As compared to *mdx* mice, which again exhibited higher plasma CK levels than WT mice (Fig. 6A), myriocin-treated mice displayed reduced CK levels, as did deflazacort-treated mice. The lowest levels of plasma CK were observed in *mdx* mice treated with the combination of myriocin and deflazacort, significantly surpassing the effects of both myriocin and deflazacort alone. We also evaluated the functional efficacy of the treatment regimens by subjecting the mice to rotarod test. Myriocin again improved performance in the rotarod test in *mdx* mice, being superior to both DMSO- and deflazacort-treated *mdx* mice (Fig. 6, B and C). The best performance in rotarod test was observed in mice receiving both myriocin and deflazacort. Improved grip strength was observed in *mdx* mice receiving deflazacort and myriocin monotherapies as well as combination therapy relative to DMSO treated *mdx* mice, the effects being similar in each of the treated groups (Fig. 6D).

To evaluate tissue morphologic changes that could underlie the improved functional performance, we analyzed histology from TA muscles of the treated groups. A modest reduction in muscle fiber size in *mdx* mice relative to WT mice was observed (Fig. 6E), as demonstrated by both CSA (Fig. 6F) and Feret diameter (Fig. 6G) in TA. This reduction was not observed in *mdx* mice treated with myriocin alone or in combination with deflazacort (Fig. 6, E to G). When quantifying the inflammatory response by the presence of the CD45^+^ pan-leukocyte marker, deflazacort, myriocin, and their combination therapy reduced the CD45^+^ inflammatory area to a similar extent in *mdx* mice (Fig. 6, H and I). Furthermore, myriocin treatment reduced fibrosis in *mdx* mice, while the combination of myriocin and deflazacort reduced fibrosis more than deflazacort alone. Our findings demonstrate that myriocin provides at least as good, if not better, fitness and morphological benefits than weekly administration of glucocorticoids.

## DISCUSSION

In the present study, we establish the involvement of sphingolipid metabolism pathway in muscular dystrophy. We demonstrate that intermediates of the sphingolipid de novo synthesis pathway accumulate in dystrophic muscle and that sphingolipid levels are elevated in the plasma of *mdx* mice. These finding are in line with the increased protein levels of enzymes of the sphingolipid de novo synthesis pathway in *mdx* muscle. Previously, sphingolipids have been associated with a number of diseases, including Alzheimer’s disease (*11*), diabetes (*13*), and cardiovascular disease (*12*). Furthermore, sphingolipids accumulate in skeletal muscle in obesity (*14*). Our discovery that sphingolipids are involved in muscular dystrophy and that the inhibition of the sphingolipid de novo synthesis pathway by myriocin provides substantial functional benefits are well in line with these studies yet substantially advancing the clinical spectrum of diseases manifesting deranged sphingolipid metabolism.

Myriocin, an inhibitor of SPT, strongly reduced the abundance of sphingolipid intermediates and reversed multiple DMD-associated, fundamental pathogenetic pathways, including aberrant Ca^2+^ homeostasis, compromised sarcolemmal membrane integrity, satellite cell imbalance, chronic inflammation, and fibrosis. The consistent beneficial effects of myriocin translated into improved functional performance of myriocin-treated mice. The observation that by inhibiting sphingolipid de novo synthesis pathway, we were able to reverse not only one but several pathophysiological pathways involved in DMD points to the high therapeutic potential of targeting sphingolipid synthesis pathway.

Myoblasts derived from patients with DMD displayed higher fold changes in sphingolipid levels relative to controls than skeletal muscle of *mdx* mice, which primarily accumulated very-long-chain sphingolipids. This observation is in agreement with the milder phenotype of *mdx* mice relative to human DMD and suggests that inhibition of sphingolipid biosynthesis might have stronger effects in human patients with DMD than in *mdx* mice.

We here focused on DMD as it is a severe, debilitating disease and the most common muscular dystrophy. Transcriptional up-regulation of sphingolipid de novo synthesis pathway was not limited to DMD but was observed in several other muscular pathologies, including EDMD and JDM, suggesting a nearly universal involvement of sphingolipid metabolism in muscular dystrophies. Thus, the use of inhibitors of sphingolipid de novo synthesis pathway could extend to diseases beyond DMD.

The pathophysiological processes of DMD, reversed by myriocin, are highly interrelated. In our study, myriocin reduced DMD-associated inflammation and attenuated M1/M2 imbalance, observed in DMD (*51*). In addition to potentially contributing to optimal function of muscle fibers (*52*, *53*), these anti-inflammatory changes, along with reduced fibrosis (*54*), could enhance satellite cell maintenance, eventually preventing their premature exhaustion (*55*). The beneficial effects of myriocin on multiple pathophysiological processes in DMD hence pave the way for detailed mechanistic follow-up studies on the particular sphingolipid classes involved, as well as tissue-specific effects of sphingolipid de novo synthesis pathway.

The observation that metabolites of the sphingolipid de novo synthesis pathway were robustly elevated in both muscle and plasma and were reduced by myriocin points to the potential role of sphingolipids as biomarkers of muscular dystrophies and to the systemic benefits of myriocin treatment, which is important given the systemic nature of DMD. In particular, very-long-chain sphingolipids could be biomarkers of the disease whose increase tails disease severity, whereas their decrease allows monitoring of therapeutic efficacy. This could be useful also when testing the efficacy of CRISPR-Cas9 gene-editing technologies.

When compared to glucocorticoids, the standard care for DMD, myriocin treatment provided additive beneficial effects. This was observed at both the levels of reduced CK levels following eccentric exercise, indicating better preserved membrane integrity, as well as improved functional performance and skeletal muscle morphology. As daily administration of glucocorticoids has been reported to cause adverse phenotypes, including fibrosis, we resorted to comparing myriocin treatment with weekly injections of glucocorticoids (*50*). While glucocorticoid dosing seems to be extremely delicate and the most optimal dosing remains yet to be found, our findings enable us to conclude that inhibition of the sphingolipid de novo synthesis itself is efficacious for dystrophic muscle and that combining a sphingolipid synthesis inhibitors with glucocorticoids could provide additional benefits relative to glucocorticoid monotherapy.

Our study aimed to uncover novel pathways underlying pathology of muscular dystrophies and potentially expose targets for treatment strategies. In addition to finding sphingolipid inhibition as a potential treatment strategy, we also showed the benefits of sphingolipid reduction on multiple biological processes downstream of dystrophin deficiency. Our study, however, is more translational than mechanistic, and the exact mechanisms by which sphingolipids are involved in DMD pathology remain to be explored in future studies. While this may sound straightforward, such an endeavor is complicated by the incomplete understanding of pathogenetic mechanisms of DMD in general. Thus, a substantial body of research is required not only to define the detailed pathogenesis of DMD but also to understand the fundamental biological processes underlying any disease with as ultimate objective to fully elucidate the involvement of sphingolipids in these diseases.

In summary, we demonstrate that sphingolipid synthesis is involved in muscular dystrophy and that its reduction by myriocin provides significant overall health benefits. The multifaceted benefits achieved by correcting aberrant sphingolipid metabolism could delay the onset of symptoms, slow the progression of disease, and ultimately counteract the loss of independence associated with muscular dystrophies. Our study presents a strong case to embark on developing novel inhibitors of the sphingolipid synthesis pathway that can be administered either alone or in combination with existing treatments.

## MATERIALS AND METHODS

### Animals

C57BL/10SnJ or C57BL/10ScSn-Dmd^mdx^J mice were purchased from the Janvier and Jackson Laboratories. Myriocin (Enzo Life Sciences, Farmingdale, NY) was first dissolved in DMSO and then mixed with phosphate-buffered saline (PBS). Three-week-old mice were treated with myriocin for 6 months at a dose of 0.4 mg/kg or with corresponding volume of DMSO three times per week. The dose was selected on the basis of previous literature showing that myriocin (0.3 mg/kg) every other day reduced atherosclerosis (*56*). For stock solution, 20 mM myriocin was dissolved in DMSO. Another cohort of 3-week-old mice was treated for 2 months with myriocin and/or deflazacort. Deflazacort [1 mg/kg intraperitoneal injections once per week] was administered as described in (*50*). All mice were housed in microisolator cages in a room illuminated from 7:00 a.m. to 7:00 p.m. with ad libitum access to normal chow diet and water. All the animal experiments are authorized by the local animal experimentation committee of the Canton de Vaud, Switzerland, under license 2890.1.

### Measurements of sphingolipids in vitro and in vivo

Cell pellets (1.2 × 10^6^ cells) and muscle tissue (20 ± 5 mg) were lysed by the addition of 100 and 200 μl of methanol (100%), respectively, spiked with the stable isotope-labeled internal standards [Spa(d17:0), Cer(d18:1/16:0)-d9, Cer(d18:1/18:0)-d7, Cer(d18:1/24:0)-d7, and Cer(d18:1/24:1)-d7)]. Sample homogenization was performed in the Cryolys Precellys Tissue Homogenizer (2 × 20 s at 10,000 rpm) (Bertin Technologies, Rockville, MD, USA) with ceramic beads. The bead beater was air-cooled down at a flow rate of 110 liters/min at 6 bars. Homogenized extracts were centrifuged for 15 min at 4000*g* at 4°C, and the resulting supernatants were collected for the liquid chromatography–mass spectrometry (LC-MS/MS) analysis. Plasma samples (20 μl) were extracted with 80 μl of methanol containing the stable isotope-labeled internal standards mentioned above. Extracts were centrifuged for 15 min at 4000*g* at 4°C, and the resulting supernatants were collected for the LC-MS/MS analysis. Sphingolipids were quantified by LC-MS/MS analysis in positive ionization mode using a 6495 triple quadrupole system (QqQ) interfaced with a 1290 UHPLC (ultrahigh-performance liquid chromatography) system (Agilent Technologies), adapted from Checa *et al.* (*57*). Briefly, the chromatographic separation was carried out in a Zorbax Eclipse plus C8 column (1.8 μm, 100 mm by 2.1 mm inside diameter) (Agilent Technologies). Mobile phase was composed of A = 5 mM ammonium formate and 0.2% formic acid in water and B = 5 mM ammonium formate and 0.2% formic acid in MeOH at a flow rate of 400 μl/min. The column temperature was 40°C, and the sample injection volume was 2 μl. The linear gradient elution starting from 80 to 100% of B (in 8 min) was applied and held until 14 min. The column was then equilibrated to initial conditions. Electrospray ionization source conditions were set as follows: a dry gas temperature of 230°C, a nebulizer of 35 psi, and a flow of 14 liters/min; and a sheath gas temperature of 400°C, a flow of 12 liters/min, a nozzle voltage of 500 V, and a capillary voltage of 4000 V. Dynamic multiple reaction monitoring was used as acquisition mode with a total cycle time of 500 ms. Optimized collision energies for each metabolite were applied. Raw LC-MS/MS data were processed using the Agilent Quantitative analysis software (version B.07.00, MassHunter, Agilent Technologies). For absolute quantification, calibration curves and the stable isotope-labeled internal standards were used to determine the response factor. Linearity of the standard curves was evaluated for each metabolite using a 12-point range. In addition, peak area integration was manually curated and corrected when necessary. Sphingolipid concentrations of cell lysates and muscle tissue were reported to protein concentrations measured in protein pellets (with bicinchoninic acid, Thermo Fisher Scientific).

### Grip strength test

Muscle strength was evaluated by a grip strength test after 5 weeks of myriocin treatment. The grip strength of each mouse for four limbs was measured on a pull-down grid assembly connected to the grip strength meter (Columbus Instruments). Each individual mouse was drawn along a straight line parallel to the grid until the grip was broken, providing the peak force in grams. This was repeated three times with 5-min intervals between each measurement.

### Endurance running

The exercise capacity of each mouse was assessed after 11 weeks of myriocin treatment by monitoring the running distance on the uphill treadmill. The exercise regimen commenced at a speed of 9 cm/s with an inclination of 5°. The speed was gradually increased 3 cm/s every 12 min. Mice were considered to be exhausted and removed from the treadmill, following the accumulation of 5 or more shocks (0.1 mA) per minute for two consecutive minutes. The distance traveled and time before exhaustion were registered as maximal running distance and period. Mice were familiarized with the treadmill 1 day before recording the running activity.

### Eccentric running and CK measurement

Mice began the exercise at a speed of 15 cm/s with a declination of 5°. The speed gradually increased after each period of 12 min of racing by 3 cm/s. The duration of each step of this protocol was 1 min. The distance traveled and the number of shocks received over 1-min intervals were recorded. All mice were set to run for 90 min except if a mouse got five shocks (0.1 mA) during two consecutive steps, which was considered exhausted and withdrawn from the running. Mice were familiarized with the treadmill 1 day before recording the running activity. Blood was collected before and immediately after the eccentric running. CK was measured on plasma collected before and after eccentric running using the Creatine Kinase Flex Reagent Cartridge (Siemens Healthcare Diagnostics AG) on the Dimension Xpand Plus Instrument (Siemens Healthcare Diagnostics AG).

### Rotarod test

The rotarod test was performed to measure muscle strength, coordination, and endurance. Mice were left undisturbed in the room for 30 min. The speed of the rotating cylinder (rotarod) increased from 0 to 40 rpm in 5 min. Each mouse experienced three trials per day for two consecutive days. The latency and speed the mouse reached at passive rotation or fall from the rotor were recorded, and the latency and speed of the best trial of the second day are presented.

### FDB muscle fiber dissociation

The FDB muscles were incubated for 38 min at 37°C in an oxygenated “Krebs-Hepes” solution [135.5 mM NaCl, 1.2 mM MgCl_2_, 5.9 mM KCl, 11.5 mM glucose, 11.5 mM Hepes, and 1.8 mM CaCl_2_ (pH 7.3) containing 0.2% collagenase type IV (Sigma-Aldrich, St. Louis, MO, USA). Muscles were then washed twice in Dulbecco’s modified Eagle’s medium/Ham’s F12 (Sigma-Aldrich) supplemented with 2% fetal bovine serum (FBS) (Sigma-Aldrich) and mechanically dissociated by repeated passages through fire-polished Pasteur pipettes of progressively decreasing diameter. Dissociated fibers were plated onto tissue culture dishes coated with Matrigel (BD Biosciences, San Jose, CA, USA) and allowed to adhere to the bottom of the dish for 2 hours. For Ca^2+^ measurements, cells were plated on glass-bottom MaTek disks. Culture dishes were kept in an incubator, with 5% CO_2_ at 37°C for 2 hours to let the fibers attach (*58*).

### Ca^2+^ imaging using Fluo-4 AM in FDB muscle fibers

FDB muscle fibers were loaded with the cytosolic Ca^2+^ indicator Fluo-4 AM (5 μM; Invitrogen, Basel, Switzerland) solubilized in a Krebs Ca^2+^ solution [135.5 mM NaCl, 1.2 mM MgCl_2_, 5.9 mM KCl, 11.5 mM glucose, 11.5 mM Hepes, and 1.8 mM CaCl_2_ (pH 7.3)] for 20 min in the incubator and then rinsed twice with Krebs solution. Immediately before image acquisition, fibers were washed twice with Ca^2+^-free Krebs solution and then kept in Ca^2+^-free Krebs solution. Fluo-4 fluorescence was monitored using a confocal microscope system (Zeiss LSM 5 Live, 40× oil-immersion lens, excitation wavelength was 488 nm, and the emitted fluorescence was recorded between 495 and 525 nm) in a time-lapse acquisition framework. After recording basal fluorescence, the fibers were stimulated with 1 μM thapsigargin (T9033, Thermo Fisher Scientific), 2.5 mM caffeine (O1728-500, Thermo Fisher Scientific), or 40 μm histamine (AC411710050, Thermo Fisher Scientific) final concentration to trigger Ca^2+^ release from the SR. A total of 2 mM CaCl_2_ was lastly used to assess SOCE. Zen software (products/microscopy-software/zenlite/zen-2-lite) was used for the acquisition, and the data were extracted to Excel files for analysis. The use of the single-excitation/emission dye Fluo-4 necessitates normalizing to prestimulation values to account for possible differences in dye loading. The amplitude of Ca^2+^ transients to indicate SR Ca^2+^ stores was calculated by subtracting the peak fluorescence from the baseline. SOCE was calculated as the difference between the calcium amplitudes after adding 2 mM CaCl_2_ and the smallest Ca^2+^ levels before adding CaCl_2_. These smallest Ca^2+^ levels before CaCl_2_ addition were used to estimate the Ca^2+^ reuptake levels (calculated as a percentage of the SR Ca^2+^ peak).

### Ex vivo muscle force assessment

Muscle mechanical measurements were assessed as previously described (*59*) with slight modifications. All assays were measured in a blinded fashion. Bl-10, *mdx*, and myriocin-treated *mdx* mice were euthanized by cervical dislocation. EDL muscles were quickly dissected and then bathed in a 10-ml horizontal chamber containing a continuously oxygenated Krebs solution composed of 135.5 mM NaCl, 5.9 mM KCl, 1 mM MgCl_2_, 2 mM CaCl_2_, 11.6 mM Hepes sodium, and 11.5 mM glucose (pH 7.4) at 25°C. The muscle was tied between a dual-mode lever arm and a fixed hook, and a stimulation was delivered through platinum electrodes running parallel to the muscle (1500A Intact Muscle Test System, Aurora Scientific Inc., Canada). Resting muscle length (*L*_0_) was carefully adjusted for maximal isometric force with 125-Hz maximally fused tetani. The force-frequency relationship was determined by sequentially stimulating the muscles at 25-, 50-, 75-, 100-, 125-, and 150-Hz stimulation trains of 300-ms duration with 1-min rest between each contraction. Normalized muscle specific force (in millinewtons per square millimeter) was expressed relative to the CSA, obtained by dividing muscle blotted weight (in milligrams) by its length and considering the fiber length equal to 0.5 *L*_0_ for EDL (*60*). To investigate muscle fatigue, muscles were subjected to 125-Hz stimulation trains of 300-ms duration at 10-s intervals over 50 s for EDL muscles. Data from each experiment were analyzed with the Aurora’s DMA software (Aurora Scientific Inc., 2002, and Solwood Enterprises Inc., 2002) and Microsoft Excel.

### Ex vivo EDL muscle eccentric contractions

The eccentric contractions were performed as previously described (*61*). Briefly, EDL muscles were subjected to a series of seven eccentric contractions consisting in 500-ms tetani during which a stretch of 1 mm was applied 160 ms after the start of stimulation and maintained up to 250 ms after the start of stimulation (10-s interval between two successive tetani). Isometric force was measured for each tetanus just before the onset of the stretch, and the percentage force drop related to the first tetanus was calculated.

### Macrophage isolation

Gastrocnemius, soleus, and quadriceps muscles from both hindlimbs were excised and transferred into PBS on ice. All muscles were trimmed, minced, and digested with Dispase II (2.5 U/ml; Roche) and 0.2% collagenase B (Roche) in PBS for 30 min at 37°C. Tissues were then centrifuged at 50*g* for 5 min, followed by the removal of the supernatant and further digestion for 20 min at 37°C twice. Muscle slurries were sequentially filtered through 100-, 70-, and 40-μm cell strainers. The isolated cells were then washed in washing buffer [Hanks’ balanced salt solution + 2.5% bovine serum albumin (BSA)] and resuspended in 800 μl of washing buffer. They were immediately stained with antibodies, including CD45 (1:200; eBioscience, eFluor450-conjugated), F4/80 (1:200; BioLegend, fluorescein isothiocyanate–conjugated), CD11b (1:200; eBioscience, phycoerythrin-cyanine7), CD11c [1:200; BD Pharmingen, allophycocyanin- eFluor 780 (APCeF780)-conjugated], and CD206 (1:200; BioLegend, eFluor-647–conjugated] for 60 min at 4°C. Secondary staining was performed with propidium iodide (PI) (Sigma-Aldrich) for 15 min at 4°C in the dark. Stained cells were analyzed using the LSRFortessa instrument (BD Biosciences). Debris and dead cells were excluded by forward scatter, side scatter, and PI gating.

### Histology

Histological specimens were prepared and analyzed as described (*62*). Muscle integrity was assessed with 1% solution of EBD, which was injected into the peritoneal cavity, using 1% volume to body weight, 24 hours before euthanasia. EBD was dissolved in PBS [0.15 M NaCl and 10 mM phosphate buffer (pH 7.4)] and sterilized by passage through membrane filters with a 0.2-μm pore size. Upon euthanasia, the hind leg skin of the mice was removed, and the animals were photographed for dye uptake into skeletal muscles, indicated by blue coloration. Muscle sections from EBD-injected or noninjected animals were then incubated in 4% paraformaldehyde at −20°C for 15 min, washed three times for 10 min with PBS, counterstained with 4′,6-diamidino-2-phenylindole (DAPI) laminin (1:200; Sigma-Aldrich), PDGFR (1:200; Cell Signaling Technology) and mounted with Dako Mounting Medium. Microscopy images of red emission fluorescence from EBD-positive muscle fibers were analyzed using the ImageJ software. Centralized nuclei percent, minimal Feret diameter, and CSA in TA muscles were determined using the ImageJ software quantification of laminin and DAPI-stained muscle images from VS120-S6-W slides scanner (Olympus). A minimum of 2000 fibers were used for each condition and measurement. The minimal Feret diameter is defined as the minimum distance between two parallel tangents at opposing borders of the muscle fiber. This measure has been found to be resistant to deviations away from the optimal cross-sectioning profile during the sectioning process.

### Treatment of BMDMs

BMDMs were isolated from the femurs and tibias of *mdx* mice by first cleaning the bones with a scalpel and 70% ethanol, followed by cutting the bones at the ends of the marrow and centrifugation at 20,000*g* for 15 min in 1.5-ml Eppendorf tubes. After removing fibroblasts by taking the supernatant after 24 hours, cells were pooled, counted, and plated at 1.6 million cells/ml in six-well plates with macrophage growth medium consisting of 85% complete RPMI 1640 [RPMI medium (Gibco), 10% FBS, and Hepes] and 15% L929-conditioned medium for 6 days. BMDMs were then stimulated with IL-4 (100 ng/ml) for 48 hours in the presence of 10 μM myriocin or corresponding volume of DMSO. A stock solution of 20 mM in DMSO was used for myriocin (Enzo). For sphingolipid measurements, four to six wells of macrophages were pooled as one replicate.

### Plasma biochemistry

For plasma biochemistry, blood samples were collected in lithium heparin–coated tubes (Microvette CB 300 Hep-Lithium, Sarstedt) from tail vein, and plasma was isolated after centrifugation at +4°C and then stored at −80°C. Plasma parameters were measured on two times diluted samples (1:1 ratio of plasma to diluent) using Dimension Xpand Plus (Siemens Healthcare Diagnostics AG, Dudingen, Switzerland). The biochemical tests were performed according to the instructions from the manufacturer’s kit for each parameter: aspartate aminotransferase (DF41A, Siemens Healthcare Diagnostics AG), alanine aminotransferase (DF143, Siemens Healthcare Diagnostics AG), and CK (DF38, Siemens Healthcare Diagnostics AG).

### Human cells

Primary human myoblast cells derived from three male healthy individuals and three male patients with DMD were provided by Hospices Civils de Lyon. The three patients with DMD and healthy individuals were all from 4- to 7-year-old males. Cells were cultured in F-10 medium supplemented with 12% FBS and penicillin/streptomycin.

### Extraction of mRNA for quantitative real-time polymerase chain reaction

Total RNA was extracted from cultured primary human myoblasts or mouse muscle tissue using TriPure reagent according to the product manual. Total RNA was transcribed to cDNA using the QuantiTect Reverse Transcription Kit (QIAGEN). Expression of selected genes was analyzed using the LightCycler 480 System (Roche) and LightCycler 480 SYBR Green I Master Reagent (Roche).

### Western blotting

DMD patient myoblast cells or mouse skeletal muscle tissues were lysed on ice in radioimmunoprecipitation assay buffer composed of 50 mM tris-HCl, 5 M NaCl, 5 mM EDTA, 0.1% sodium dodecyl sulfate (SDS), 100 mM NaF, sodium deoxycholate (5 mg/ml), and 1% NP-40 containing protease and phosphatase inhibitors (Roche). Protein concentrations were determined using the Bradford method, and samples were loaded on a 12% SDS–polyacrylamide gel electrophoresis (PAGE) gel. After electrophoresis, proteins were separated by SDS-PAGE and transferred onto methanol-activated polyvinylidene difluoride membranes. Blocking of the membranes was done in 5% milk-TBST (tris-buffered saline with 0.1% Tween 20) for 1 hour, and after washing, the membranes were incubated overnight with primary antibody anti-SPTLC1 (Proteintech) or anti-SPTLC2 (Thermo Fisher Scientific) or anti-CERS2 (Sigma-Aldrich) in 3% BSA-TBST (1:1000). Incubation with secondary anti-rabbit polyclonal antibody was done in 5% BSA-TBST (1:2000). Antibody detection reactions were developed by enhanced chemiluminescence (Advansta) and imaged using the c300 Imaging System (Azure Biosystems).

### Transcriptome of human muscle biopsies

The muscle biopsies from different human muscular dystrophies are described in E-GEOD-3307 (*16*). The biopsies obtained from presymptomatic patients with DMD (E-GEOD-6011) (*18*) were obtained during the first 2 years of their life with age-matched controls. The biopsies from symptomatic patients with DMD are described in E-GEOD-38417; the age of the controls is unknown.

### Statistics

Heatmaps were generated using the heatmap2 function of glops R package. *z* scores were used to construct heatmaps. In the exploratory analysis in Fig. 1B, asterisks are based on Benjamini-Hochberg false discovery rate (BH FDR)–adjusted *P* values provided by E-GEOD-3307 (https://ebi.ac.uk/gxa/experiments/E-GEOD-3307/Results), which control the FDRs over the full sets of genes tested in each dataset. In the confirmation analyses using datasets E-GEOD-38417 (symptomatic DMD; Fig. 1B) and E-GEOD-6011 (presymptomatic DMD; Fig. 1C), asterisks denoting statistical significance are based on BH FDR–adjusted *P* values from Student’s two-tailed *t* tests performed on the raw data.

The following genes were used in principal components analysis in E-GEOD-38417 human biopsies (Affymetrix GeneChip HG-U133_Plus_2; Fig. 4, B and C): *SPTLC1*, *SPTLC2*, *KDSR*, *CERS1*, *CERS2*, *CERS5*, *CERS6*, and *DEGS1*. For GSEA using GO categories, transcripts were ordered according to their Pearson correlation, and 10,000 permutations were used. BH FDR–adjusted *P* < 0.05 was considered significant. The principal components analyses of the “GO: positive regulation of immune response” and “GO: innate immune response” categories were performed by including all annotated genes in the respective pathways. The nominal *P* values and Pearson *r* for Fig. 4 (B and C) were obtained by correlating the first PC of GO: positive regulation of immune response and GO: innate immune response pathways with the first PC of *SphPC1*.

For comparison of the effects of DMSO, deflazacort, myriocin, and the combination of myriocin and deflazacort in *mdx* mice in Fig. 6 (A to D, F, G, I, and K), one-way analysis of variance (ANOVA) with Tukey’s post hoc testing was performed. As heteroscedasticity was observed for CK measurements (Levene’s test, *P* < 0.01) in Fig. 6A, statistical analysis was performed using square root–transformed values of CK. Untransformed CK measurements were plotted to facilitate visualization. For the other phenotypes, untransformed experimental measurements were used for statistical analyses. Tukey-corrected *P* < 0.05 was considered significant.

In all other cases, comparisons were done between WT versus *mdx* mice and *mdx* mice versus *mdx* treated with myriocin with Student’s two-tailed *t* tests on the raw data, and the BH FDR was controlled per figure panel, except for Figs. 1 (F to H, J to L, and M to O) and 2 (D to F and G to I), where the BH FDR correction was done in each tissue for all sphingolipid species (sphinganine, dihydroceramides, and ceramides) (i.e., the FDR was controlled each time over three figure panels). For quantitative polymerase chain reaction results, *P* values were calculated using Student’s *t* test with log-transformed expression values with BH FDR adjustment. In all analyses, adjusted *P* < 0.05 was considered significant.

## Notes

View/request a protocol for this paper from *Bio-protocol*.
