# Supplementary material for: Inhibition of sphingolipid de novo synthesis counteracts muscular dystrophy
Source: Sci Adv. 2022 Jan 28;8(4):eabh4423. doi: 10.1126/sciadv.abh4423 (PMC8797791; doi:10.1126/sciadv.abh4423)
Supplement: Supplementary file 1 — Figs. S1 to S5 Table S2 Legend for table S1 [file sciadv.abh4423_sm.pdf]

Science Advances
AAAS

Supplementary Materials for

Inhibition of sphingolipid de novo synthesis counteracts muscular dystrophy

Pirkka-Pekka Laurila, Peiling Luan, Martin Wohlwend, Nadège Zanou, Barbara Crisol, Tanes Imamura de Lima, Ludger J. E. Goeminne, Hector Gallart-Ayala, Minho Shong, Julijana Ivanisevic, Nicolas Place, Johan Auwerx*

*Corresponding author. Email: admin.auwerx@epfl.ch

Published 28 January 2022, *Sci. Adv.* **8**, eabh4423 (2022)
DOI: 10.1126/sciadv.abh4423

The PDF file includes:

Figs. S1 to S5
Table S2
Legend for table S1

Other Supplementary Material for this manuscript includes the following:

Table S1

Supplementary figures

Figure S1. Sphinganine (left), dihydroceramide (middle), and ceramide (right) levels in quadriceps muscle of (**A**) 4 week old and (**B**) 10 week old C57/BL10 and *mdx* mice.

Figure S2. (**A**) Proportion of variance explained by each principal component of the sphingolipid *de novo* synthesis pathway in skeletal muscle of symptomatic DMD patients and controls (E-GEOD-38417). Levels of alanine aminotransferase (**B**) and aspartate aminotransferase (**C**) in WT mice, *mdx* mice, and *mdx* mice treated with myriocin. (**D**) Quantification of developed force at 25s (middle) of the fatigue protocol in EDL muscle of mice. Results are expressed relative to the developed force at the 1st tetanus. (**E**) Quantification of force drop during the 50s fatigue protocol in EDL muscle from WT (C57/BL10), *mdx*, and *mdx* mice treated with myriocin (force quantified every 10th tetanus). (**F**) Quantification of force drop during eccentric EDL muscle contractions in WT (C57/BL10), *mdx*, and *mdx* mice treated with myriocin. (**G**) Representative traces of cytosolic Ca^{2+} transients in isolated FDB muscle fibers upon 2.5 mM caffeine stimulation (SR Ca^{2+} store) and after 2 mM $CaCl_2$ (SOCE). (**H**) Ca^{2+} amplitude (SR Ca^{2+} store) upon 2.5 mM caffeine stimulation as percentage of C57/BL10. (**I**) Ca^{2+} amplitude (SR Ca^{2+} store) upon 40 µM histamine stimulation as percentage of C57/BL10. (**J**) Sarcoplasmic reticulum Ca^{2+} uptake after 2.5 mM caffeine stimulation as percentage of Ca^{2+} peak. All data are shown as mean ± SEM. Statistical significance is calculated using Student's two-tailed T test with BH adjustment for FDR. *BH FDR < 0.05, **BH FDR < 0.01, and ***BH FDR < 0.001.

Figure S3. Sphinganine (**A**), dihydroceramide (**B**), and ceramide (**C**) levels in primary bone marrow derived macrophages of C57/BL10 or *mdx* mice. For **A-C**, statistical significance is calculated using Student's two-tailed T test with BH adjustment for FDR. All data are shown as mean ± SEM. *BH FDR < 0.05, **BH FDR < 0.01, and ***BH FDR < 0.001.

Figure S4. Sphinganine (left), dihydroceramide (middle), and ceramide (right) levels in (**A**) diaphragm and (**B**) heart tissue of C57/BL10 or *mdx* mice. For **A-B**, statistical significance is calculated using Student's two-tailed T test with BH adjustment for FDR. All data are shown as mean ± SEM. *BH FDR < 0.05, **BH FDR < 0.01, and ***BH FDR < 0.001.

Figure S5. (**A**) Immunostaining of PDGFR, a marker of FAPs, and DAPI in diaphragm. Scale bar, 50 µm. (**B**) Immunostaining of PDGFR, and DAPI in TA. Scale bar, 50 µm.

Figure S1

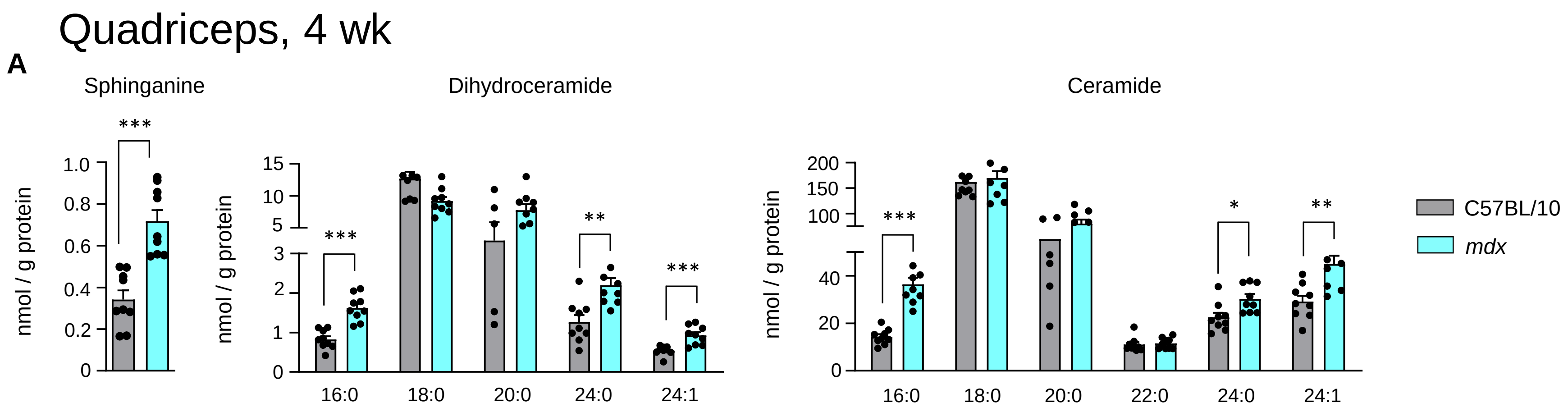
A
Quadriceps, 4 wk
Sphinganine
Dihydroceramide
Ceramide
nmol / g protein
***
**
*
16:0
18:0
20:0
22:0
24:0
24:1
C57BL/10
mdx

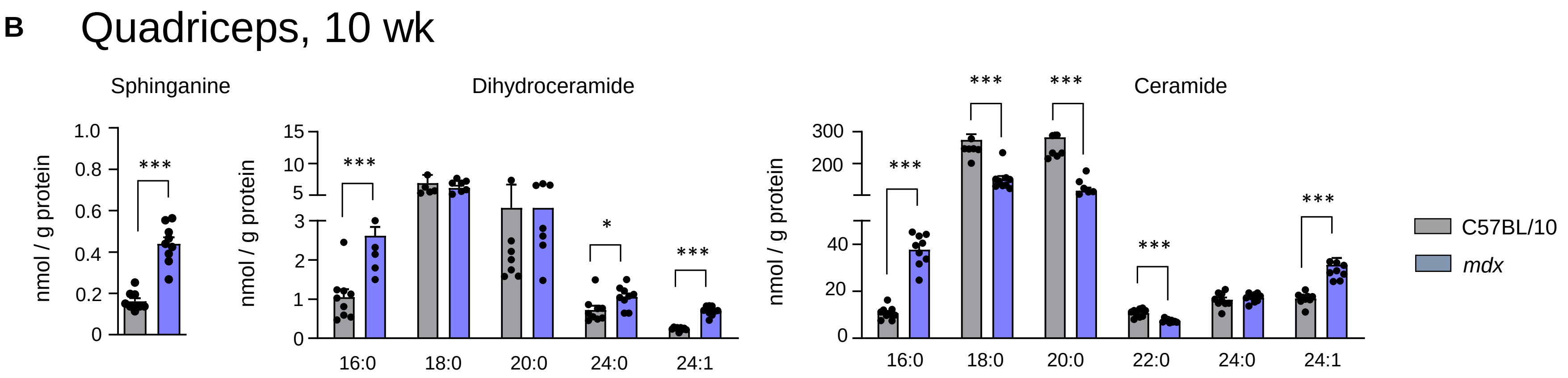
B
Quadriceps, 10 wk
Sphinganine
Dihydroceramide
Ceramide
nmol / g protein
***
*
16:0
18:0
20:0
22:0
24:0
24:1
C57BL/10
mdx

Figure S2

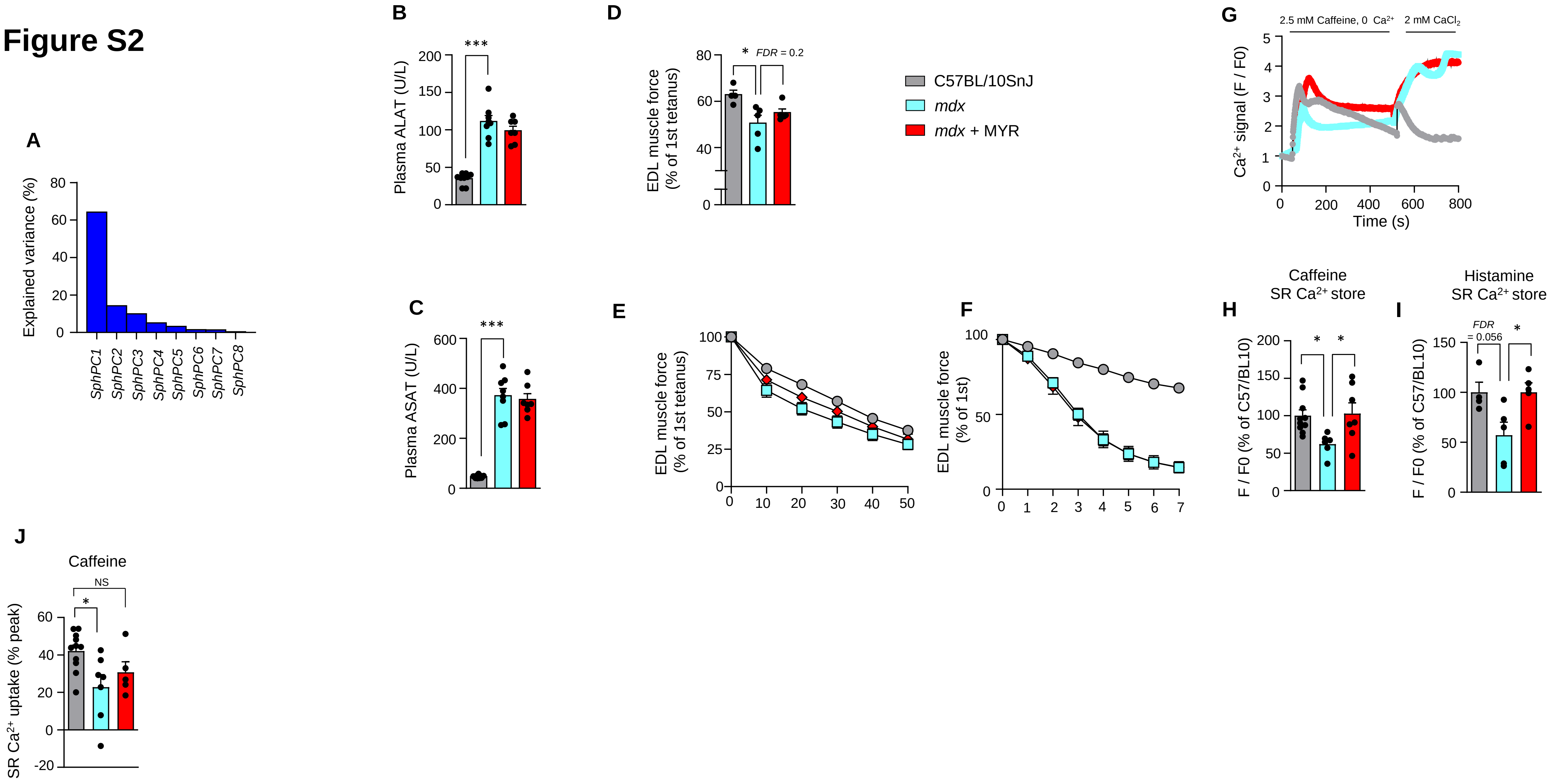
A
Explained variance (%)
SphPC1
SphPC2
SphPC3
SphPC4
SphPC5
SphPC6
SphPC7
SphPC8
B
Plasma ALAT (U/L)
***
C
Plasma ASAT (U/L)
***
D
EDL muscle force (% of 1st tetanus)
*
FDR = 0.2
C57BL/10SnJ
mdx
mdx + MYR
E
EDL muscle force (% of 1st tetanus)
F
EDL muscle force (% of 1st)
G
2.5 mM Caffeine, 0 Ca2+
2 mM CaCl2
Ca2+ signal (F / F0)
Time (s)
Caffeine SR Ca2+ store
H
F / F0 (% of C57/BL10)
Histamine SR Ca2+ store
I
F / F0 (% of C57/BL10)
FDR = 0.056
J
Caffeine
NS
SR Ca2+ uptake (% peak)

Figure S3

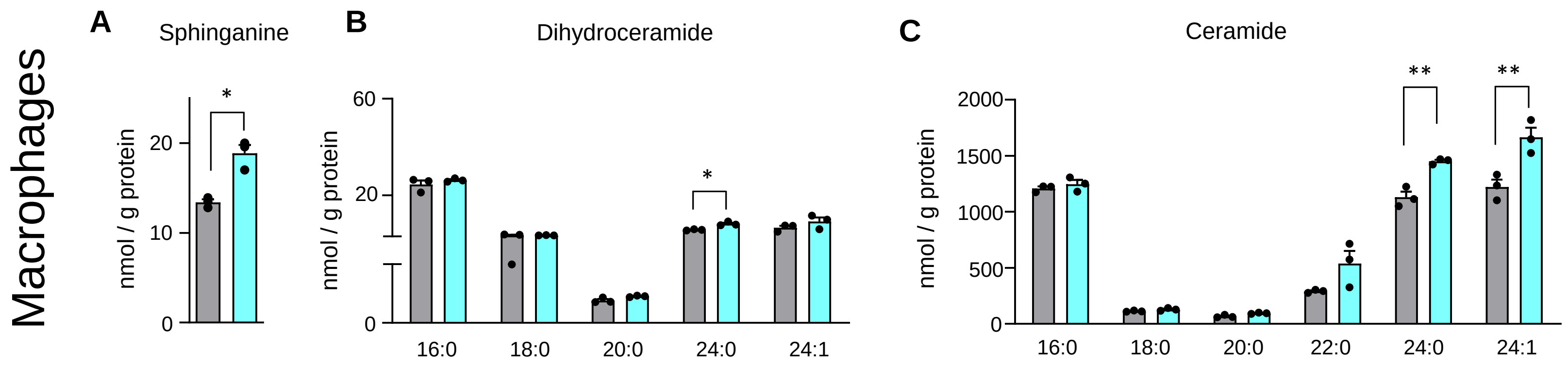
Macrophages
A
Sphinganine
nmol / g protein
0
10
20
*
B
Dihydroceramide
nmol / g protein
0
20
60
*
16:0
18:0
20:0
24:0
24:1
C
Ceramide
nmol / g protein
0
500
1000
1500
2000
**
**
16:0
18:0
20:0
22:0
24:0
24:1

Figure S4

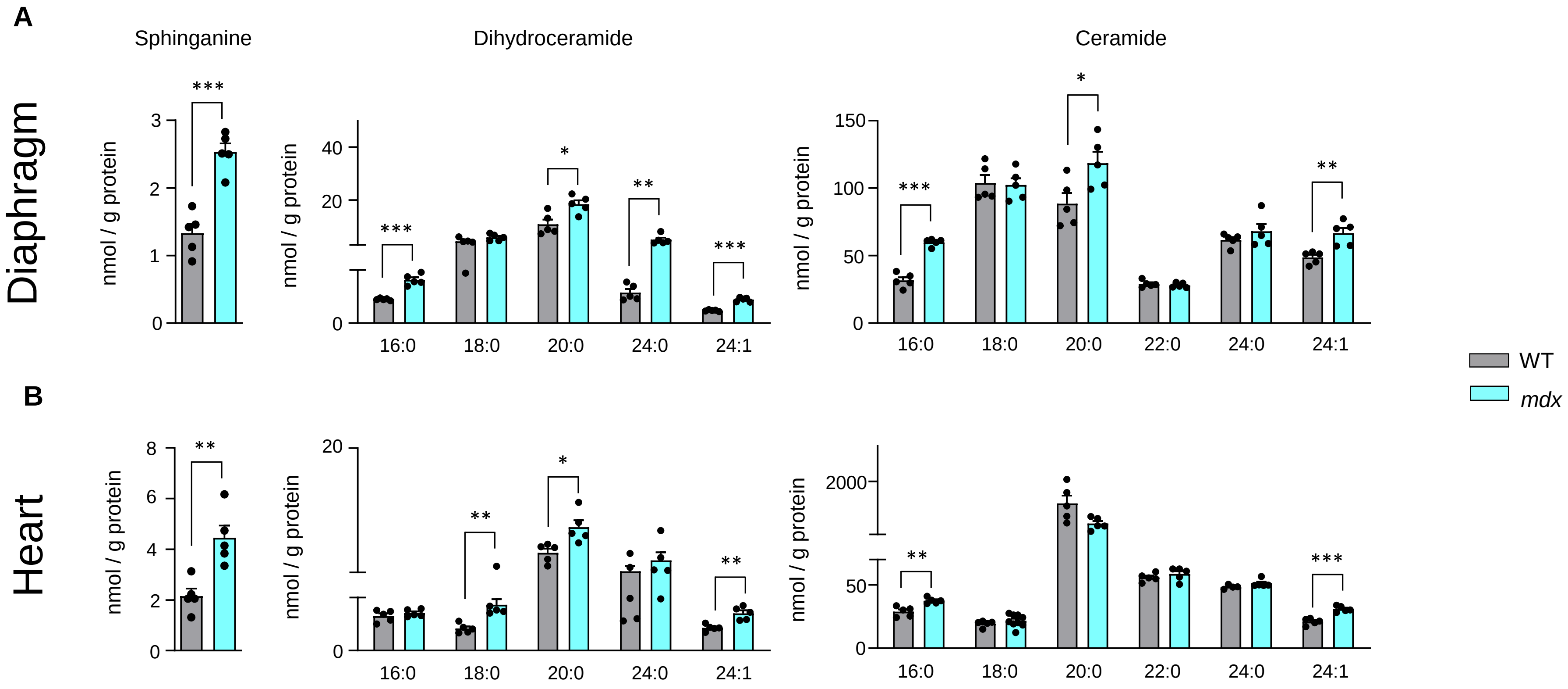
A
Sphinganine
Dihydroceramide
Ceramide
Diaphragm
nmol / g protein
***
*
**
16:0
18:0
20:0
22:0
24:0
24:1
WT
mdx
B
Heart

Figure S5

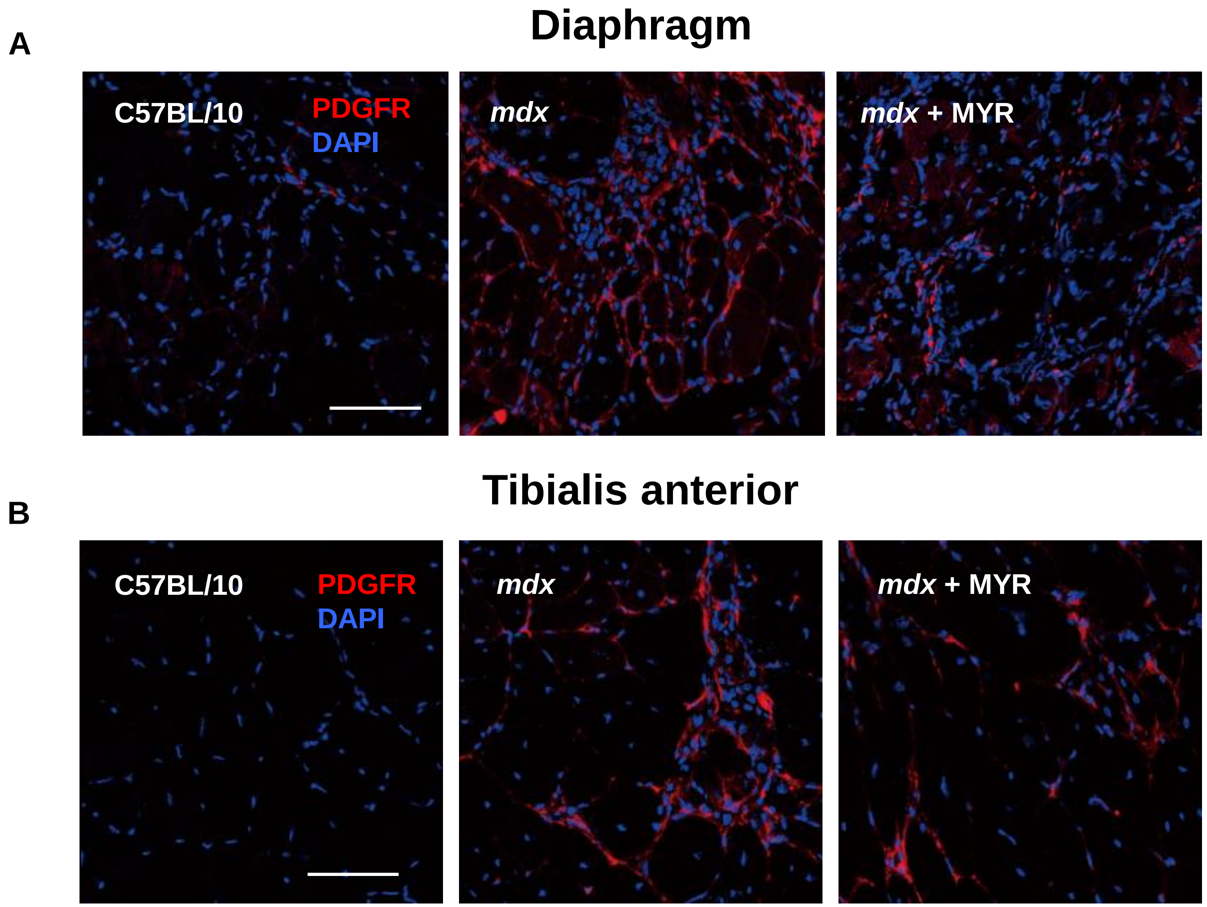
A
Diaphragm
C57BL/10
PDGFR
DAPI
mdx
mdx + MYR
B
Tibialis anterior
C57BL/10
PDGFR
DAPI
mdx
mdx + MYR

Supplementary Table 1. Pathways correlated with the first principal component of sphingolipid *de novo* synthesis pathway (please see separate file).

Supplementary Table 2. List of primers.

**Gene symbol (mouse)**	**Forward**	**Reverse**
Cd163	TCCACACGTCCAGAACAGTC	CCTTGGAAACAGAGACAGGC
Retnla	ACCTTTCCTGAGATTCTGCCCC	CAGTGGTCCAGTCAACGAGTAAGC
Il10	TGAATTCCCTGGGTGAGAAGCTGA	TGGCCTTGTAGACACCTTGGTCTT
Il6	GCCTTCTTGGGACTGATGCT	TGCCATTGCACAACTCTTTTCT
Il1b	TGCCATTGCACAACTCTTTTCT	GGTGGAGAGCTTTCAGCTCATAT
iNOS	CCCTTCAATGGTTGGTACATGG	ACATTGATCTCCGTGACAGCC
B2m	TTCTGGTGCTTGTCTCACTG	TATGTTCGGCTTCCCATTCT
