# Supplementary material for: Inhibition of sphingolipid de novo synthesis counteracts muscular dystrophy
Source: Sci Adv. 2022 Jan 28;8(4):eabh4423. doi: 10.1126/sciadv.abh4423 (PMC8797791; doi:10.1126/sciadv.abh4423)
Supplement: Supplementary file 2 — Table S1 [file sciadv.abh4423_table_s1.zip › sciadv.abh4423_table_s1.pdf]

Supplementary Table 1

ID	setSize	NES	pvalue	p.adjust
GO_POSITIVE_REGULATION_OF_IMMUNE_RESPONSE	468	1.921548953	0.000114116	0.007857378
GO_CELL_CELL_ADHESION	468	1.893971498	0.000114116	0.007857378
GO_CELL_ACTIVATION	463	1.948827663	0.000114482	0.007857378
GO_INNATE_IMMUNE_RESPONSE	444	2.340925456	0.000114784	0.007857378
GO_RESPONSE_TO_WOUNDING	455	1.855142296	0.000114718	0.007857378
GO_VASCULATURE_DEVELOPMENT	419	1.741164894	0.000115647	0.007857378
GO_SKELETAL_SYSTEM_DEVELOPMENT	392	1.904201069	0.000116877	0.007857378
GO_IMMUNE_EFFECTOR_PROCESS	386	2.433513278	0.000117275	0.007857378
GO_SINGLE_ORGANISM_CELL_ADHESION	380	2.073420659	0.000117523	0.007857378
GO_WOUND_HEALING	376	1.904713773	0.000117633	0.007857378
GO_TAXIS	364	1.841486475	0.000118427	0.007857378
GO_ACTIVATION_OF_IMMUNE_RESPONSE	357	1.868818486	0.000118483	0.007857378
GO_DEFENSE_RESPONSE_TO_OTHER_ORGANISM	343	1.839785487	0.000118835	0.007857378
GO_LEUKOCYTE_ACTIVATION	340	1.90074097	0.00011946	0.007857378
GO_REGULATION_OF_RESPONSE_TO_WOUNDING	336	1.900626694	0.000119632	0.007857378
GO_BLOOD_VESSEL_MORPHOGENESIS	324	1.865342552	0.00012025	0.007857378
GO_SMALL_GTPASE_MEDIATED_SIGNAL_TRANSDUCTION	321	1.718837095	0.000120744	0.007857378
GO_NEGATIVE_REGULATION_OF_IMMUNE_SYSTEM_PROCESS	310	2.046142039	0.000121359	0.007857378
GO_REGULATION_OF_CELL_CELL_ADHESION	310	1.687386995	0.000121359	0.007857378
GO_REGULATION_OF_INNATE_IMMUNE_RESPONSE	305	1.755211347	0.000122026	0.007857378
GO_LYMPHOCYTE_ACTIVATION	279	1.88711943	0.000122926	0.007857378
GO_EXTRACELLULAR_STRUCTURE_ORGANIZATION	263	2.283337682	0.000124162	0.007857378
GO_ANGIOGENESIS	263	1.903262138	0.000124162	0.007857378
GO_POSITIVE_REGULATION_OF_CELL_ACTIVATION	254	1.736012546	0.000124797	0.007857378
GO_VIRAL_LIFE_CYCLE	251	1.916601813	0.000125172	0.007857378
GO_HEMOSTASIS	242	1.741996444	0.000125834	0.007857378
GO_POSITIVE_REGULATION_OF_RESPONSE_TO_EXTERNAL_STIMULUS	245	1.815004208	0.00012604	0.007857378
GO_REGULATION_OF_INFLAMMATORY_RESPONSE	240	1.748156645	0.00012631	0.007857378
GO_CELL_PROJECTION_ASSEMBLY	227	2.114846366	0.00012734	0.007857378
GO_LEUKOCYTE_MIGRATION	219	1.939730457	0.000128123	0.007857378
GO_OSSIFICATION	216	2.062284693	0.000128271	0.007857378
GO_LEUKOCYTE_CELL_CELL_ADHESION	207	1.95855715	0.000128436	0.007857378
GO_ADAPTIVE_IMMUNE_RESPONSE	198	2.174890728	0.000129483	0.007857378
GO_RESPONSE_TO_VIRUS	200	2.15147685	0.00012982	0.007857378
GO_RNA_CATABOLIC_PROCESS	196	2.059320323	0.000130191	0.007857378
GO_REGULATED_EXOCYTOSIS	187	1.735740265	0.000130497	0.007857378
GO_NEGATIVE_REGULATION_OF_CELL_ADHESION	184	1.948449167	0.000130976	0.007857378
GO_LYMPHOCYTE_DIFFERENTIATION	167	1.764045099	0.000132205	0.007857378
GO_CILIUM_MORPHOGENESIS	173	2.00436458	0.00013224	0.007857378
GO_CONNECTIVE_TISSUE_DEVELOPMENT	165	1.786503802	0.000132837	0.007857378
GO_CILIUM_ORGANIZATION	156	1.890179297	0.000133654	0.007857378
GO_CELL_SUBSTRATE_ADHESION	150	1.899932571	0.000134228	0.007857378
GO_CARBOHYDRATE_DERIVATIVE_CATABOLIC_PROCESS	153	1.923705215	0.000134481	0.007857378
GO_AMINOGLYCAN_METABOLIC_PROCESS	143	1.93377944	0.000135593	0.007857378
GO_BONE_DEVELOPMENT	139	2.265014921	0.000135648	0.007857378
GO_LEUKOCYTE_MEDIATED_IMMUNITY	138	2.022446304	0.000135704	0.007857378
GO_PROTEIN_TARGETING_TO_MEMBRANE	132	2.405427384	0.000136537	0.007857378
GO_NEGATIVE_REGULATION_OF_CELL_ACTIVATION	132	1.994090758	0.000136537	0.007857378

GO_DEFENSE_RESPONSE_TO_VIRUS	129	2.410135595	0.000136649	0.007857378
GO_REGULATION_OF_CELL_SHAPE	125	2.039783286	0.000137231	0.007857378
GO_REGULATION_OF_LEUKOCYTE_MIGRATION	125	1.779865343	0.000137231	0.007857378
GO_CARTILAGE_DEVELOPMENT	123	1.867736382	0.000137344	0.007857378
GO_TRANSLATIONAL_INITIATION	120	2.135353677	0.000137552	0.007857378
GO_PLATELET_ACTIVATION	120	1.90069665	0.000137552	0.007857378
GO_RESPONSE_TO_INTERFERON_GAMMA	115	2.212660331	0.000138274	0.007857378
GO_REGULATION_OF_CYTOKINE_SECRETION	115	2.14702096	0.000138274	0.007857378
GO_MULTI_ORGANISM_METABOLIC_PROCESS	117	2.37591456	0.000138351	0.007857378
GO_ADAPTIVE_IMMUNE_RESPONSE_BASED_ON_SOMATIC_RECOMBINATION_OF_IMN	112	1.822599825	0.000138504	0.007857378
GO_HUMORAL_IMMUNE_RESPONSE	108	1.844849675	0.000139684	0.007857378
GO_PROTEIN_LOCALIZATION_TO_ENDOPLASMIC_RETICULUM	104	2.925089164	0.000139919	0.007857378
GO_NEGATIVE_REGULATION_OF_IMMUNE_RESPONSE	103	2.030253273	0.000140331	0.007857378
GO_CELL_ACTIVATION_INVOLVED_IN_IMMUNE_RESPONSE	105	2.03104704	0.000140726	0.007857378
GO_LYMPHOCYTE_MEDIATED_IMMUNITY	102	1.999029597	0.000140726	0.007857378
GO_CYTOKINE_PRODUCTION	105	1.810983716	0.000140726	0.007857378
GO_NUCLEAR_TRANSCRIBED_MRNA_CATABOLIC_PROCESS_NONSENSE_MEDIATED_DE	99	2.651361201	0.000140825	0.007857378
GO_PLATELET_DEGRANULATION	95	1.939230811	0.000141844	0.007857378
GO_CELLULAR_RESPONSE_TO_INTERFERON_GAMMA	96	2.255078475	0.000142025	0.007857378
GO_AMINOGLYCAN_BIOSYNTHETIC_PROCESS	93	1.979426932	0.000142308	0.007857378
GO_ESTABLISHMENT_OF_PROTEIN_LOCALIZATION_TO_ENDOPLASMIC_RETICULUM	85	2.910742303	0.000143575	0.007857378
GO_MULTICELLULAR_ORGANISM_METABOLIC_PROCESS	76	2.155828884	0.000145243	0.007857378
GO_MULTICELLULAR_ORGANISMAL_MACROMOLECULE_METABOLIC_PROCESS	64	2.474369708	0.000148987	0.007857378
GO_B_CELL_MEDIATED_IMMUNITY	63	2.069121966	0.000149254	0.007857378
GO_INTERFERON_GAMMA_MEDIATED_SIGNALING_PATHWAY	61	2.458379686	0.000149522	0.007857378
GO_EXTRACELLULAR_MATRIX_DISASSEMBLY	61	1.988287433	0.000149522	0.007857378
GO_AMINOGLYCAN_CATABOLIC_PROCESS	57	2.129824722	0.000150534	0.007857378
GO_RESPONSE_TO_TYPE_I_INTERFERON	53	2.715428701	0.000151103	0.007857378
GO_PROTEOGLYCAN_BIOSYNTHETIC_PROCESS	51	2.159367322	0.000151492	0.007857378
GO_COLLAGEN_FIBRIL_ORGANIZATION	35	2.336479942	0.000158153	0.007857378
GO_CYTOKINE_SECRETION	34	2.031561088	0.000158428	0.007857378
GO_CHONDROITIN_SULFATE_PROTEOGLYCAN_BIOSYNTHETIC_PROCESS	26	2.324928253	0.000160668	0.007857378
GO_CHONDROITIN_SULFATE_BIOSYNTHETIC_PROCESS	22	2.407881395	0.000161421	0.007857378
GO_RESPONSE_TO_INTERFERON_ALPHA	19	2.197107381	0.000162787	0.007857378
GO_RAC_PROTEIN_SIGNAL_TRANSDUCTION	18	2.247309057	0.000164799	0.007857378
GO_RESPONSE_TO_PLATELET_DERIVED_GROWTH_FACTOR	18	2.103546103	0.000164799	0.007857378
GO_CELLULAR_RESPONSE_TO_CYTOKINE_STIMULUS	491	1.608059473	0.000227118	0.010319452
GO_REGULATION_OF_CELL_MORPHOGENESIS	480	1.586583386	0.000228076	0.010319452
GO_REGULATION_OF_CYTOKINE_PRODUCTION	467	1.726488476	0.000228519	0.010319452
GO_ENDOCYTOSIS	444	1.662811237	0.000229568	0.010319452
GO_REGULATION_OF_CELL_ACTIVATION	396	1.708842427	0.000232883	0.010319452
GO_RESPONSE_TO_BACTERIUM	387	1.651327085	0.000234577	0.010319452
GO_POSITIVE_REGULATION_OF_LOCOMOTION	364	1.671896694	0.000236855	0.010319452
GO_CYTOKINE_MEDIATED_SIGNALING_PATHWAY	353	1.741170737	0.000237051	0.010319452
GO_REGULATION_OF_HOMOTYPIC_CELL_CELL_ADHESION	252	1.718021027	0.00024975	0.010623568
GO_LEUKOCYTE_DIFFERENTIATION	241	1.713888812	0.000252175	0.010623568
GO_NEGATIVE_REGULATION_OF_LOCOMOTION	228	1.711452985	0.000254647	0.010623568
GO_PHAGOCYTOSIS	159	1.793691448	0.000267487	0.010672657
GO_REGULATION_OF_OSSIFICATION	151	1.745391738	0.000268492	0.010672657
GO_REGULATION_OF_CHEMOTAXIS	152	1.707724136	0.000268745	0.010672657

GO_RAS_PROTEIN_SIGNAL_TRANSDUCTION	139	1.806470165	0.000271297	0.010672657
GO_MYELOID_LEUKOCYTE_ACTIVATION	84	1.899473212	0.000287274	0.010734014
GO_NEGATIVE_REGULATION_OF_VIRAL_PROCESS	77	1.959173165	0.000290107	0.010734014
GO_FC_GAMMA_RECEPTOR_SIGNALING_PATHWAY	76	1.895745516	0.000290487	0.010734014
GO_PROTEOGLYCAN_METABOLIC_PROCESS	73	2.064776199	0.000292612	0.010734014
GO_BONE_MORPHOGENESIS	66	2.080085436	0.000297929	0.010734014
GO_NEGATIVE_REGULATION_OF_VIRAL_GENOME_REPLICATION	44	1.946700344	0.00030774	0.010734014
GO_CHONDROITIN_SULFATE_PROTEOGLYCAN_METABOLIC_PROCESS	39	2.079229501	0.00030955	0.010734014
GO_REGULATION_OF_TOLL_LIKE_RECEPTOR_SIGNALING_PATHWAY	41	1.96380942	0.000310078	0.010734014
GO_REGULATION_OF_MAST_CELL_ACTIVATION	37	2.076961306	0.000313578	0.010734014
GO_HUMORAL_IMMUNE_RESPONSE_MEDIATED_BY_CIRCULATING_IMMUNOGLOBULI	37	2.037902869	0.000313578	0.010734014
GO_POSITIVE_REGULATION_OF_MYELOID_LEUKOCYTE_MEDIATED_IMMUNITY	15	2.067255039	0.000334113	0.010959601
GO_NEGATIVE_REGULATION_OF_NATURAL_KILLER_CELL_MEDIATED_IMMUNITY	11	2.19849506	0.000345006	0.011048266
GO_REGULATION_OF_BODY_FLUID_LEVELS	394	1.576330707	0.000350345	0.011048266
GO_POSITIVE_REGULATION_OF_CELL_ADHESION	318	1.660774139	0.000362976	0.011198725
GO_POSITIVE_REGULATION_OF_DEFENSE_RESPONSE	313	1.652249961	0.000363504	0.011198725
GO_ESTABLISHMENT_OF_PROTEIN_LOCALIZATION_TO_MEMBRANE	233	1.606776479	0.000379939	0.011491966
GO_RRNA_METABOLIC_PROCESS	229	1.66400912	0.000381631	0.011491966
GO_REGULATION_OF_LEUKOCYTE_PROLIFERATION	169	1.662690509	0.000395361	0.011816576
GO_CELLULAR_RESPONSE_TO_ACID_CHEMICAL	149	1.697094048	0.000404968	0.011818273
GO_REGULATION_OF_LEUKOCYTE_MEDIATED_IMMUNITY	137	1.783099539	0.000407221	0.011818273
GO_NEGATIVE_REGULATION_OF_MULTI_ORGANISM_PROCESS	124	1.760435832	0.000412031	0.011871838
GO_REGULATION_OF_OSTEOBLAST_DIFFERENTIATION	99	1.855466007	0.000422476	0.012020421
GO_B_CELL_ACTIVATION	97	1.881104327	0.000423191	0.012020421
GO_LYMPHOCYTE_ACTIVATION_INVOLVED_IN_IMMUNE_RESPONSE	66	1.936473315	0.000446894	0.012317165
GO_PROTEIN_LOCALIZATION_TO_ORGANELLE	493	1.51175851	0.000453515	0.012317165
GO_IMMUNE_SYSTEM_DEVELOPMENT	485	1.534281662	0.000455166	0.012317165
GO_CELL_MORPHOGENESIS_INVOLVED_IN_DIFFERENTIATION	417	1.607876229	0.000463124	0.012336362
GO_REGULATION_OF_LEUKOCYTE_DEGRANULATION	39	2.035479132	0.000464324	0.012336362
GO_NEGATIVE_REGULATION_OF_LEUKOCYTE_MEDIATED_IMMUNITY	41	1.956052627	0.000465116	0.012336362
GO_INFLAMMATORY_RESPONSE	356	1.571421705	0.000474271	0.012414736
GO_CHONDROCYTE_DEVELOPMENT	20	2.035146465	0.000488759	0.012689511
GO_RESPONSE_TO_LIPOPROTEIN_PARTICLE	18	2.008580406	0.000494397	0.012689511
GO_MAST_CELL_ACTIVATION	18	2.000553361	0.000494397	0.012689511
GO_REGULATION_OF_VASCULATURE_DEVELOPMENT	203	1.633457516	0.000516996	0.012875661
GO_NEURON_PROJECTION_GUIDANCE	156	1.693311082	0.000534616	0.013209974
GO_NEGATIVE_REGULATION_OF_CELLULAR_RESPONSE_TO_GROWTH_FACTOR_STIMU	99	1.829647392	0.000563301	0.013449696
GO_NEGATIVE_REGULATION_OF_IMMUNE_EFFECTOR_PROCESS	91	1.842701882	0.00056899	0.013449696
GO_MUCOPOLYSACCHARIDE_METABOLIC_PROCESS	94	1.805573654	0.00056899	0.013449696
GO_RESPONSE_TO_GROWTH_FACTOR	419	1.567283104	0.000578235	0.013449696
GO_CELL_DIVISION	412	1.566517832	0.000580248	0.013449696
GO_TISSUE_MIGRATION	76	1.834347926	0.000580973	0.013449696
GO_REGULATION_OF_IMMUNE_EFFECTOR_PROCESS	357	1.571990252	0.000592417	0.013557888
GO_EXOCYTOSIS	268	1.610933128	0.000617742	0.013899185
GO_REGULATION_OF_MONONUCLEAR_CELL_MIGRATION	14	2.031415945	0.000672043	0.014826661
GO_POSITIVE_REGULATION_OF_IMMUNE_EFFECTOR_PROCESS	134	1.750309423	0.000680921	0.014826661
GO_RESPONSE_TO_IONIZING_RADIATION	133	1.73635243	0.000681849	0.014826661
GO_TRANSMEMBRANE_RECEPTOR_PROTEIN_TYROSINE_KINASE_SIGNALING_PATHWA	445	1.516575829	0.000688468	0.014826661
GO_KERATAN_SULFATE_CATABOLIC_PROCESS	11	2.091647745	0.000690012	0.014826661
GO_NEGATIVE_REGULATION_OF_CELL_CELL_ADHESION	113	1.741729994	0.000691181	0.014826661

GO_REGULATION_OF_PROTEIN_SERINE_THREONINE_KINASE_ACTIVITY	428	1.517173906	0.000692281	0.014826661
GO_RETINA_DEVELOPMENT_IN_CAMERA_TYPE_EYE	99	1.812749378	0.000704126	0.015000135
GO_REGULATION_OF_TUMOR_NECROSIS_FACTOR_SUPERFAMILY_CYTOKINE_PRODUC	89	1.827093319	0.000711744	0.01500281
GO_RESPONSE_TO_VITAMIN	80	1.786687092	0.000726639	0.015192553
GO_REGULATION_OF_VIRAL_GENOME_REPLICATION	70	1.875332948	0.000737137	0.015296547
GO_POSITIVE_REGULATION_OF_OSTEOBLAST_DIFFERENTIATION	53	1.941488459	0.000755515	0.015517121
GO_COMPLEMENT_ACTIVATION	44	1.892115304	0.000769349	0.015720629
GO_SUBSTRATE_ADHESION_DEPENDENT_CELL_SPREADING	37	1.924207938	0.000783945	0.01578059
GO_REGULATION_OF_MAST_CELL_ACTIVATION_INVOLVED_IN_IMMUNE_RESPONSE	30	2.036385693	0.000795925	0.01578059
GO_REGULATION_OF_LYMPHOCYTE_MIGRATION	30	1.981261273	0.000795925	0.01578059
GO_TUBE_DEVELOPMENT	466	1.512231835	0.000800183	0.01578686
GO_REGULATION_OF_EXTRINSIC_APOPTOTIC_SIGNALING_PATHWAY	134	1.718475105	0.000817105	0.015885944
GO_PROTEIN_LOCALIZATION_TO_MEMBRANE	336	1.57001533	0.000837421	0.016202271
GO_MATURE_B_CELL_DIFFERENTIATION_INVOLVED_IN_IMMUNE_RESPONSE	11	2.046895146	0.000862515	0.0165281
GO_POSITIVE_REGULATION_OF_CYTOKINE_SECRETION	75	1.837411213	0.000873617	0.016582157
GO_B_CELL_DIFFERENTIATION	63	1.888250468	0.000895522	0.01691777
GO_POSITIVE_REGULATION_OF_CELL_CELL_ADHESION	199	1.641295678	0.000906266	0.017011016
GO_CHONDROCYTE_DIFFERENTIATION	51	1.927281948	0.000908953	0.017011016
GO_ORGANIC_CYCLIC_COMPOUND_CATABOLIC_PROCESS	362	1.529009472	0.000947082	0.017503868
GO_NEGATIVE_REGULATION_OF_INNATE_IMMUNE_RESPONSE	35	1.911085948	0.000948917	0.017503868
GO_CELL_CHEMOTAXIS	135	1.693972845	0.00095277	0.017503868
GO_REGULATION_OF_DENDRITIC_CELL_DIFFERENTIATION	12	2.010858474	0.001020235	0.018657716
GO_MEMBRANE_LIPID_METABOLIC_PROCESS	158	1.635083507	0.001071524	0.019330877
GO_RESPONSE_TO_TRANSFORMING_GROWTH_FACTOR_BETA	134	1.684951548	0.001089473	0.019526171
GO_POSITIVE_REGULATION_OF_CYTOKINE_PRODUCTION	309	1.552357086	0.0010921	0.019526171
GO_POSITIVE_REGULATION_OF_CHEMOTAXIS	103	1.760352126	0.001122649	0.019855828
GO_REGULATION_OF_EPITHELIAL_CELL_PROLIFERATION	249	1.554273747	0.001130369	0.019855828
GO_CELLULAR_RESPONSE_TO_VITAMIN	20	2.007489139	0.001140437	0.019906309
GO_NEGATIVE_REGULATION_OF_CELL_KILLING	16	1.935286654	0.001177856	0.020245985
GO_REGULATION_OF_PHAGOCYTOSIS	56	1.908239705	0.001203732	0.020538146
GO_MITOTIC_NUCLEAR_DIVISION	322	1.535334393	0.00120511	0.020538146
GO_RESPONSE_TO_MOLECULE_OF_BACTERIAL_ORIGIN	280	1.565532524	0.00122835	0.020788847
GO_CELLULAR_RESPONSE_TO_AMINO_ACID_STIMULUS	45	1.908575083	0.001230201	0.020788847
GO_PROTEIN_TRIMERIZATION	34	1.903601255	0.001267427	0.021238685
GO_HEMATOPOIETIC_PROGENITOR_CELL_DIFFERENTIATION	82	1.758040666	0.001298514	0.021641878
GO_REGULATION_OF_NON_CANONICAL_WNT_SIGNALING_PATHWAY	19	1.963732337	0.001302295	0.021641878
GO_NEGATIVE_REGULATION_OF_CYTOKINE_PRODUCTION	176	1.61661526	0.001315616	0.021772907
GO_GLYCOPROTEIN_METABOLIC_PROCESS	289	1.571952584	0.001346719	0.022195923
GO_POSITIVE_REGULATION_OF_RESPONSE_TO_WOUNDING	130	1.689570543	0.00136612	0.022423408
GO_REGULATION_OF_T_CELL_PROLIFERATION	119	1.700486825	0.001372495	0.022436095
GO_ANTIGEN_PROCESSING_AND_PRESENTATION	185	1.620223005	0.001437345	0.023370758
GO_NEGATIVE_REGULATION_OF_MITOTIC_CELL_CYCLE	176	1.610291411	0.001447178	0.023370758
GO_NEURAL_TUBE_DEVELOPMENT	127	1.691904065	0.001508916	0.02417284
GO_REGULATION_OF_CELLULAR_RESPONSE_TO_GROWTH_FACTOR_STIMULUS	190	1.608727144	0.001568627	0.025029295
GO_REGULATION_OF_MAP_KINASE_ACTIVITY	289	1.552999809	0.001591577	0.025294704
GO_POSITIVE_REGULATION_OF_OSSIFICATION	74	1.778789238	0.001608893	0.025441494
GO_POSITIVE_REGULATION_OF_KINASE_ACTIVITY	426	1.469214343	0.001614577	0.025441494
GO_REGULATION_OF_EPITHELIAL_CELL_MIGRATION	149	1.63888111	0.00161987	0.025441494
GO_MEMBRANE_RAFT_ORGANIZATION	17	1.927149776	0.001662234	0.025903686
GO_DERMATAN_SULFATE_PROTEOGLYCAN_METABOLIC_PROCESS	15	1.933885623	0.001670565	0.025932602

GO_REGULATION_OF_CELL_ADHESION_MEDIATED_BY_INTEGRIN	35	1.853447942	0.001739681	0.026804753
GO_SECRETION_BY_CELL	403	1.478065112	0.001746827	0.026804753
GO_NEURON_PROJECTION_MORPHOGENESIS	314	1.523418456	0.001820167	0.027823552
GO_POSITIVE_REGULATION_OF_MAPK_CASCADE	390	1.492618765	0.001871126	0.028483767
GO_EYE_DEVELOPMENT	251	1.533349159	0.001877582	0.028483767
GO_SUBSTRATE_DEPENDENT_CELL_MIGRATION	25	1.949500608	0.001923077	0.028911018
GO_PROTEIN_LOCALIZATION_TO_CILIUM	23	1.89785449	0.001927401	0.028911018
GO_REGULATION_OF_WOUND_HEALING	105	1.698372594	0.001970166	0.02944222
GO_PROTEIN_TARGETING	359	1.507298813	0.002015412	0.029895278
GO_ANTIGEN_PROCESSING_AND_PRESENTATION_OF_PEPTIDE_ANTIGEN	157	1.600562452	0.002275161	0.033500067
GO_NEURON_PROJECTION_DEVELOPMENT	438	1.45073259	0.002410745	0.034982007
GO_NEGATIVE_REGULATION_OF_HOMOTYPIC_CELL_CELL_ADHESION	84	1.728134887	0.002441827	0.035206439
GO_CYTOLYSIS	20	1.878190977	0.002443793	0.035206439
GO_LEUKOCYTE_CHEMOTAXIS	93	1.697425727	0.002561548	0.036770613
GO_CELLULAR_RESPONSE_TO_LIPOPROTEIN_PARTICLE_STIMULUS	11	1.964355233	0.002587545	0.037011139
GO_EPHRIN_RECEPTOR_SIGNALING_PATHWAY	78	1.719774919	0.002609452	0.037191656
GO_MYELOID_CELL_ACTIVATION_INVOLVED_IN_IMMUNE_RESPONSE	39	1.793425768	0.002631172	0.037368236
GO_PROTEIN_ACTIVATION_CASCADE	59	1.788526309	0.00270027	0.03807728
GO_NEGATIVE_REGULATION_OF_LEUKOCYTE_PROLIFERATION	57	1.818181675	0.002709619	0.03807728
GO_POSITIVE_REGULATION_OF_PEPTIDASE_ACTIVITY	141	1.613094672	0.002847458	0.039735428
GO_REGULATION_OF_ANTIGEN_RECEPTOR_MEDIATED_SIGNALING_PATHWAY	33	1.826731025	0.002858958	0.039757386
GO_POSITIVE_REGULATION_OF_EXTRINSIC_APOPTOTIC_SIGNALING_PATHWAY	49	1.808667079	0.002903423	0.040236017
GO_T_CELL_DIFFERENTIATION	105	1.676803995	0.002955249	0.040813009
GO_NEGATIVE_REGULATION_OF_CYTOKINE_SECRETION	35	1.817598107	0.003004903	0.041073841
GO_REGULATION_OF_EXTRINSIC_APOPTOTIC_SIGNALING_PATHWAY_VIA_DEATH_DOI	52	1.808360033	0.003022518	0.041119448
GO_POSITIVE_REGULATION_OF_LEUKOCYTE_DEGRANULATION	16	1.862543195	0.003028773	0.041119448
GO_NEGATIVE_REGULATION_OF_CELL_DIFFERENTIATION	479	1.419479992	0.003080434	0.041679515
GO_GLOMERULUS_DEVELOPMENT	42	1.804996662	0.003114295	0.041995791
GO_NEGATIVE_REGULATION_OF_CELL_DEVELOPMENT	246	1.505506082	0.00315179	0.042348943
GO_DIGESTIVE_SYSTEM_DEVELOPMENT	118	1.627378607	0.003167608	0.042348943
GO_ENDOTHELIAL_CELL_MIGRATION	54	1.791889937	0.003172205	0.042348943
GO_POSITIVE_REGULATION_OF_I_KAPPAB_KINASE_NF_KAPPAB_SIGNALING	165	1.591484589	0.003188098	0.042419706
GO_NEGATIVE_REGULATION_OF_EXOCYTOSIS	25	1.866171294	0.003205128	0.042422063
GO_ANTIGEN_PROCESSING_AND_PRESENTATION_OF_ENDOGENOUS_PEPTIDE_ANTIGI	13	1.921396932	0.003209459	0.042422063
GO_NEGATIVE_REGULATION_OF_LEUKOCYTE_APOPTOTIC_PROCESS	40	1.780084062	0.003244747	0.042747402
GO_POSITIVE_REGULATION_OF_MAST_CELL_ACTIVATION	15	1.882508416	0.003341129	0.043729486
GO_SKELETAL_SYSTEM_MORPHOGENESIS	171	1.566634057	0.003435518	0.044818402
GO_CYTOKINESIS	76	1.699691879	0.003485839	0.045244477
GO_NEGATIVE_REGULATION_OF_LYMPHOCYTE_MEDIATED_IMMUNITY	30	1.812856004	0.003502069	0.045244477
GO_RHYTHMIC_PROCESS	240	1.497924472	0.003536693	0.045496825
GO_POSITIVE_REGULATION_OF_INFLAMMATORY_RESPONSE	90	1.685245326	0.003555682	0.045496825
GO_REPRODUCTIVE_BEHAVIOR	18	1.844256676	0.003625577	0.045950744
GO_MICROVILLUS_ORGANIZATION	18	1.843185209	0.003625577	0.045950744
GO_POSITIVE_REGULATION_OF_TOLL_LIKE_RECEPTOR_SIGNALING_PATHWAY	18	1.827165135	0.003625577	0.045950744
GO_B_CELL_ACTIVATION_INVOLVED_IN_IMMUNE_RESPONSE	28	1.84580538	0.003675296	0.046287921
GO_REGULATION_OF_DNA_DAMAGE_RESPONSE_SIGNAL_TRANSDUCTION_BY_P53_CL	28	1.824222212	0.003675296	0.046287921
GO_POSITIVE_REGULATION_OF_ACUTE_INFLAMMATORY_RESPONSE	23	1.851609603	0.003694186	0.04637998
GO_REGULATION_OF_PEPTIDASE_ACTIVITY	301	1.473221551	0.003770372	0.047041559
GO_NEGATIVE_REGULATION_OF_INTERLEUKIN_12_PRODUCTION	13	1.880475304	0.003885135	0.048322876
GO_REGULATION_OF_ADAPTIVE_IMMUNE_RESPONSE	105	1.64434707	0.003940332	0.048857678

GO_POSITIVE_REGULATION_OF_INNATE_IMMUNE_RESPONSE	220	1.51842444	0.003968762	0.048924439
GO_REGULATION_OF_MEMBRANE_LIPID_DISTRIBUTION	29	1.816549623	0.003981526	0.048924439
GO_NEGATIVE_REGULATION_OF_CELL_CYCLE_G1_S_PHASE_TRANSITION	90	1.67064367	0.003982364	0.048924439
GO_NEGATIVE_REGULATION_OF_DEFENSE_RESPONSE	122	1.608610079	0.003995591	0.048936826
GO_CELL_CYCLE_CHECKPOINT	172	1.550277814	0.004091329	0.04980478
GO_DETECTION_OF_MECHANICAL_STIMULUS_INVOLVED_IN_SENSORY_PERCEPTION_(	10	-2.011312284	0.004017963	0.049060794
GO_STRIATED_MUSCLE_CONTRACTION	92	-1.631849646	0.003721245	0.046573706
GO_GLUCAN_METABOLIC_PROCESS	52	-1.792831113	0.003545052	0.045496825
GO_PORPHYRIN_CONTAINING_COMPOUND_METABOLIC_PROCESS	31	-1.918602248	0.003495563	0.045244477
GO_REGULATION_OF_TRANSPORTER_ACTIVITY	169	-1.537817367	0.003314002	0.043516645
GO_PROTEIN_HOMOTETRAMERIZATION	55	-1.815828713	0.002985075	0.040942548
GO_PROTEIN_K63_LINKED_UBIQUITINATION	32	-1.96113739	0.002974581	0.040938819
GO_REGULATION_OF_DOPAMINE_SECRETION	15	-2.090890545	0.002739044	0.038356191
GO_AMINO_ACID_ACTIVATION	52	-1.870795193	0.002658789	0.037627028
GO_GLYOXYLATE_METABOLIC_PROCESS	23	-2.03197564	0.002383475	0.034712057
GO_MITOCHONDRIAL_FISSION	11	-2.100823263	0.002378121	0.034712057
GO_POSITIVE_REGULATION_OF_MITOCHONDRION_ORGANIZATION	145	-1.610024644	0.002299732	0.033737822
GO_STEROID_HORMONE_MEDIATED_SIGNALING_PATHWAY	111	-1.668838155	0.002130682	0.03148849
GO_ORGANELLE_DISASSEMBLY	163	-1.620296805	0.002007226	0.029884536
GO_REGULATION_OF_CELLULAR_KETONE_METABOLIC_PROCESS	148	-1.628511345	0.001923077	0.028911018
GO_REGULATION_OF_OXIDATIVE_PHOSPHORYLATION	15	-2.201279203	0.001743028	0.026804753
GO_MONOCARBOXYLIC_ACID_CATABOLIC_PROCESS	83	-1.827725164	0.001627604	0.025463104
GO_PYRUVATE_METABOLIC_PROCESS	52	-1.952248652	0.001477105	0.023758253
GO_MUSCLE_CELL_DEVELOPMENT	113	-1.723765374	0.001445087	0.023370758
GO_ORGANIC_ACID_CATABOLIC_PROCESS	176	-1.604106989	0.001249479	0.021025903
GO_TETRAPYRROLE_METABOLIC_PROCESS	49	-2.02192085	0.001156738	0.019968688
GO_COFACTOR_BIOSYNTHETIC_PROCESS	146	-1.670508737	0.001149425	0.019928348
GO_NAD_METABOLIC_PROCESS	45	-1.964191482	0.001143184	0.019906309
GO_MUSCLE_FIBER_DEVELOPMENT	39	-1.991657594	0.001129624	0.019855828
GO_MONOVALENT_INORGANIC_CATION_TRANSPORT	304	-1.504558698	0.001111729	0.019788772
GO_SMALL_MOLECULE_CATABOLIC_PROCESS	276	-1.579171312	0.001066098	0.019320013
GO_PROTEIN_MODIFICATION_BY_SMALL_PROTEIN_REMOVAL	105	-1.810354518	0.001035912	0.0188583
GO_CRISTAE_FORMATION	11	-2.242017027	0.000951249	0.017503868
GO_PROTEIN_TRANSMEMBRANE_TRANSPORT	48	-2.064234013	0.000866802	0.016531143
GO_REGULATION_OF_HEART_CONTRACTION	189	-1.682521223	0.000855432	0.016471179
GO_PEPTIDE_METABOLIC_PROCESS	484	-1.57680036	0.000815661	0.015885944
GO_NUCLEOBASE_CONTAINING_SMALL_MOLECULE_METABOLIC_PROCESS	470	-1.756346294	0.000814332	0.015885944
GO_TETRAPYRROLE_BIOSYNTHETIC_PROCESS	26	-2.165255084	0.000794071	0.01578059
GO_CELLULAR_COMPONENT_DISASSEMBLY	453	-1.71334343	0.000784929	0.01578059
GO_AMIDE_BIOSYNTHETIC_PROCESS	442	-1.550726754	0.000773994	0.015735255
GO_MITOCHONDRIAL_ATP_SYNTHESIS_COUPLED_PROTON_TRANSPORT	17	-2.340320637	0.000752634	0.015517121
GO_INORGANIC_ION_TRANSMEMBRANE_TRANSPORT	414	-1.480239297	0.000728332	0.015192553
GO_HYDROGEN_TRANSPORT	109	-1.834574372	0.000708466	0.01500281
GO_OXIDOREDUCTION_COENZYME_METABOLIC_PROCESS	89	-1.856522189	0.000671817	0.014826661
GO_PURINE_CONTAINING_COMPOUND_METABOLIC_PROCESS	348	-2.043920391	0.000626566	0.014018986
GO_FATTY_ACID_CATABOLIC_PROCESS	67	-1.93306124	0.000614251	0.013898722
GO_AUTOPHAGY	339	-1.774235083	0.000610501	0.013892358
GO_GLYCOSYL_COMPOUND_METABOLIC_PROCESS	323	-2.060937354	0.000588582	0.013547523
GO_PROTEIN_TARGETING_TO_MITOCHONDRION	46	-2.089339761	0.000573888	0.013449696
GO_RIBONUCLEOSIDE_TRIPHOSPHATE_BIOSYNTHETIC_PROCESS	41	-2.143241994	0.000563063	0.013449696

GO_CELLULAR_PROTEIN_COMPLEX_ASSEMBLY	299	-1.714591442	0.000556483	0.013449696
GO_COFACTOR_METABOLIC_PROCESS	287	-1.732096105	0.000545256	0.013315557
GO_NADH_METABOLIC_PROCESS	30	-2.223218507	0.000537634	0.013209974
GO_ENERGY_COUPLED_PROTON_TRANSPORT_DOWN_ELECTROCHEMICAL_GRADIENT	20	-2.243151102	0.000517598	0.012875661
GO_GENERATION_OF_PRECURSOR_METABOLITES_AND_ENERGY	257	-3.045934514	0.000503778	0.012689511
GO_MUSCLE_SYSTEM_PROCESS	255	-1.806604713	0.000500751	0.012689511
GO_MITOCHONDRIAL_ELECTRON_TRANSPORT_CYTOCHROME_C_TO_OXYGEN	15	-2.309138627	0.000498008	0.012689511
GO_MACROAUTOPHAGY	235	-1.66763758	0.000472144	0.012414736
GO_NUCLEOSIDE_MONOPHOSPHATE_METABOLIC_PROCESS	215	-2.426440224	0.000452489	0.012317165
GO_MUSCLE_CONTRACTION	210	-1.950097745	0.000447828	0.012317165
GO_NUCLEOSIDE_TRIPHOSPHATE_METABOLIC_PROCESS	202	-2.530390571	0.000439754	0.01231618
GO_ENERGY_DERIVATION_BY_OXIDATION_OF_ORGANIC_COMPOUNDS	197	-3.17881009	0.000434972	0.012268041
GO_MACROMOLECULAR_COMPLEX_DISASSEMBLY	165	-2.475953763	0.000404204	0.011818273
GO_MITOCHONDRIAL_TRANSPORT	161	-2.276419127	0.000398883	0.011818273
GO_CELLULAR_RESPIRATION	133	-3.541752317	0.000374672	0.011454672
GO_CELLULAR_PROTEIN_COMPLEX_DISASSEMBLY	111	-2.776833378	0.000355114	0.011111173
GO_TRANSLATIONAL_ELONGATION	104	-3.095045085	0.000350263	0.011048266
GO_MITOCHONDRIAL_TRANSLATION	102	-3.379541597	0.000345304	0.011048266
GO_MITOCHONDRIAL_MEMBRANE_ORGANIZATION	90	-2.066934264	0.000336587	0.010959601
GO_TRANSLATIONAL_TERMINATION	88	-3.219691817	0.000332557	0.010959601
GO_ELECTRON_TRANSPORT_CHAIN	88	-3.363779109	0.000332557	0.010959601
GO_HYDROGEN_ION_TRANSMEMBRANE_TRANSPORT	82	-2.335935144	0.000325627	0.010959122
GO_OXIDATIVE_PHOSPHORYLATION	78	-3.406291058	0.000322165	0.010934497
GO_PROTEIN_LOCALIZATION_TO_MITOCHONDRION	64	-2.309001247	0.000303951	0.010734014
GO_MITOCHONDRIAL_RESPIRATORY_CHAIN_COMPLEX_ASSEMBLY	64	-3.11852708	0.000303951	0.010734014
GO_MITOCHONDRIAL_TRANSMEMBRANE_TRANSPORT	51	-2.61222153	0.000294031	0.010734014
GO_MITOCHONDRIAL_RESPIRATORY_CHAIN_COMPLEX_I_BIOGENESIS	51	-3.108145441	0.000294031	0.010734014
GO_AEROBIC_RESPIRATION	48	-2.899985644	0.000288934	0.010734014
GO_TRICARBOXYLIC_ACID_METABOLIC_PROCESS	35	-2.501746943	0.000271813	0.010672657
GO_ATP_BIOSYNTHETIC_PROCESS	29	-2.349897607	0.000268601	0.010672657
GO_INNER_MITOCHONDRIAL_MEMBRANE_ORGANIZATION	17	-2.513140608	0.000250878	0.010623568
